# Comparative Effects of CuNPs, CuONPs and CuSO_4_ on Biomass Quality, Culture Liquid Composition and Biostimulant Activity of *Nostoc linckia*

**DOI:** 10.3390/life16081302

**Published:** 2026-08-08

**Authors:** Liliana Cepoi, Tatiana Chiriac, Ludmila Rudi, Svetlana Codreanu, Ana Valuța, Svetlana Djur, Tudor Trifan, Vera Potopová

**Affiliations:** 1 Institute of Microbiology and Biotechnology, Technical University of Moldova, 2028 Chisinau, Moldova; liliana.cepoi@imb.utm.md (L.C.); tatiana.chiriac@imb.utm.md (T.C.); svetlana.codreanu@imb.utm.md (S.C.); ana.valuta@imb.utm.md (A.V.); svetlana.djur@imb.utm.md (S.D.); 2 Faculty of Agrobiology, Food and Natural Resources, Department of Agroecology and Crop Production, Czech University of Life Sciences Prague, Kamýcká 129, Praha-Suchdol, 165 00 Prague, Czech Republic; trifan@af.czu.cz (T.T.); potop@af.czu.cz (V.P.)

**Keywords:** *Nostoc linckia*, copper form, biomass quality, culture liquid composition, biostimulant activity, maize seed priming

## Abstract

The chemical form of copper is a key determinant of metal bioavailability and may profoundly influence cyanobacterial metabolism and the biological activity of culture-derived products. In this study, *Nostoc linckia* was cultivated in the presence of metallic copper nanoparticles (CuNPs), copper oxide nanoparticles (CuONPs), or CuSO_4_·5H_2_O (15 mg Cu L^−1^). Biomass productivity, biochemical composition, and antioxidant capacity, together with the physicochemical and biochemical characteristics of the culture liquid, were determined. The culture liquid was subsequently evaluated through the seed priming of four maize (*Zea mays* L.) hybrids. The different copper forms induced distinct metabolic responses in *Nostoc linckia*. CuONPs promoted biomass accumulation while maintaining protein and photosynthetic pigment contents and inducing an adaptive oxidative response, whereas CuNPs, and particularly CuSO_4_, impaired biomass production and primary metabolism. These metabolic changes were reflected in the culture liquid properties and were associated with genotype-dependent differences in maize germination dynamics, seedling vigor, and biochemical composition. Integrated correlation, hierarchical clustering, and principal component analyses revealed coordinated relationships linking cyanobacterial physiology, culture liquid profiles, and plant responses. Collectively, these findings demonstrate that the chemical form of copper drives metabolic remodeling in *Nostoc linckia*, thereby shaping the biochemical composition and biostimulant properties of its culture liquid.

## 1. Introduction

In recent decades, photosynthetic microorganisms have emerged as some of the most valuable biological resources for the development of a circular bioeconomy. This is due to their ability to sustainably produce biomass, capture carbon dioxide, utilize renewable resources, and generate a wide range of value-added compounds for agriculture, the food and pharmaceutical industries, and environmental protection [1,2,3,4,5,6,7]. In agriculture, these microorganisms are used as natural biofertilizers and biostimulants capable of promoting seed germination, plant growth and development, nutrient uptake, and tolerance to environmental stresses, thereby enhancing the productivity and sustainability of agricultural systems [1,4,5,8,9,10,11].

Among the cyanobacteria with biotechnological potential, members of the order Nostocales, and particularly *Nostoc linckia*, are distinguished by their remarkable metabolic plasticity, ability to fix atmospheric nitrogen, and capacity to synthesize a broad spectrum of bioactive compounds, including photosynthetic pigments, proteins, extracellular polysaccharides, lipids, and antioxidant metabolites. These characteristics, together with their high tolerance to various environmental stresses, including metal exposure, make *Nostoc linckia* a particularly suitable model for the present study. Its pronounced metabolic plasticity enables the modulation of extracellular metabolite production in response to cultivation conditions, allowing the resulting culture liquid to serve as an informative system for investigating how different copper forms influence cyanobacterial metabolism and the biostimulant properties of extracellular products. These features also make *Nostoc linckia* a promising candidate for applications in agriculture, bioremediation, and the production of value-added bioproducts [4,12,13,14,15,16,17].

The suitability of *Nostoc linckia* for this purpose is further supported by the high responsiveness of its metabolism to cultivation conditions. The biochemical composition and biological activity of *Nostoc linckia* biomass and its derived products, as well as those of other cyanobacteria, are strongly influenced by cultivation conditions. Consequently, the development of strategies to precisely modulate cellular metabolism has become a major research priority in phycobiotechnology. Carefully controlled abiotic stressors can induce adaptive responses and stimulate the biosynthesis of metabolites with high biological and agronomic value without compromising culture viability. Within this context, research has progressively shifted from maximizing biomass production toward the controlled modulation of cellular metabolism to obtain products with enhanced functional properties. One effective strategy involves the use of elicitors, physical or chemical factors that modulate microbial metabolism and induce the differential synthesis of metabolites involved in adaptive responses, as well as the biological activity of the resulting products. Among these, metallic compounds of essential elements are of particular interest because, depending on their chemical form and concentration, they can simultaneously function as micronutrients and as inducers of adaptive physiological and biochemical responses [18,19,20].

Copper is an essential trace element involved in the functioning of the photosynthetic apparatus, electron transport, and numerous metalloenzymes [20]. At elevated concentrations, excess copper disrupts redox homeostasis, promotes the generation of reactive oxygen species, and triggers the reorganization of cellular metabolism. In contrast, at sublethal levels, copper-induced stress can activate adaptive mechanisms and stimulate the accumulation of protective metabolites with significant biotechnological value, consistent with the principle of hormesis—an adaptive response to low levels of stress or damage resulting in improved fitness for some physiological systems for a finite period [21]. In photosynthetic microorganisms, hormetic responses may include the stimulation of growth, preservation of photosynthetic pigments, enhanced metabolic activity, and activation of antioxidant defense systems. These responses are generally attributed to the activation of cellular stress-response pathways that improve tolerance to moderate stress. From this perspective, copper represents a promising elicitor for the controlled modulation of cyanobacterial metabolism.

Recent advances in nanotechnology have significantly expanded the potential applications of copper as an elicitor by enabling the development of nano-structured forms with physicochemical properties distinct from those of conventional metal salts. Copper nanoparticles (CuNPs) and copper oxide nanoparticles (CuONPs) differ from copper ions released from soluble salts such as CuSO_4_ in particle size, specific surface area, and ion-release kinetics, thereby influencing bioavailability and the cellular responses of photosynthetic microorganisms [20,22,23].

The cultivation of *Nostoc linckia* yields two complementary biological fractions: cellular biomass, the principal product used in biotechnological applications, and the culture liquid, which contains extracellular polysaccharides, proteins, amino acids, organic acids, redox-active compounds, signaling molecules, and other extracellular metabolites. Its composition depends on the physiological characteristics of the microorganism, cultivation conditions, and the intensity of the applied stress factors [24,25,26]. Although cyanobacterial biomass has been extensively exploited as a source of biostimulants and biofertilizers, the culture liquid remains insufficiently characterized from biochemical and functional perspectives despite growing evidence demonstrating that extracellular metabolites can stimulate seed germination, plant growth, and tolerance to abiotic stress [5,24,26,27]. The integrated valorization of both biomass and culture liquid aligns with the principles of the circular bioeconomy and the biorefinery concept, promoting the full utilization of biological streams generated during biotechnological processes.

Based on the hypothesis that the chemical form of copper differentially modulates the metabolism of *Nostoc linckia*, thereby influencing biomass quality, the composition of the culture liquid, and consequently its biological activity toward plants, the aim of the present study was to comparatively evaluate the effects of copper nanoparticles (CuNPs), copper oxide nanoparticles (CuONPs), and copper sulfate pentahydrate (CuSO_4_·5H_2_O) on the quality of *Nostoc linckia* biomass, characterize the biochemical and physicochemical changes occurring in the culture liquid, and assess its biostimulant potential on the germination and early seedling development of maize (*Zea mays* L.). This integrated approach addresses the underexplored link between copper form, culture liquid remodeling, and plant-related bioactivity, thereby providing a scientific basis for the development of next-generation biostimulants.

## 2. Materials and Methods

### 2.1. Copper Compounds

Three copper forms were used in the experiments: metallic copper nanoparticles (CuNPs), copper oxide nanoparticles (CuONPs), and copper sulfate pentahydrate (CuSO_4_·5H_2_O). The nanoparticles and copper sulfate pentahydrate were purchased from Sigma-Aldrich (Merck KGaA, Darmstadt, Germany). According to the manufacturer’s specifications, as determined by transmission electron microscopy (TEM), the average particle sizes were 25 nm for the CuNPs and 50 nm for the CuONPs. Stock suspensions of nanoparticles were freshly prepared by dispersing the nanoparticle powders in deionized water, followed by ultrasonic treatment for 25 min under temperature-controlled conditions (at 20 °C). The freshly prepared suspensions were immediately added to the culture medium to obtain the desired final copper concentration.

### 2.2. Determination of Dissolved Copper

Stock suspensions of CuNPs, CuONPs, and CuSO_4_ (1 mg Cu mL^−1^) were prepared in sterile deionized water. The suspensions were sonicated in an ultrasonic bath (SONOREX DIGITEC DT, BANDELIN electronic GmbH & Co. KG, Berlin, Germany; 35 kHz) for 25 min at 20 °C and vortex-mixed for 30 s immediately before use. Appropriate volumes were transferred into sterile mineral medium to obtain a final copper concentration of 15 mg Cu L^−1^. The suspensions were incubated under the same physicochemical conditions used for the cultivation of *Nostoc linckia* (25 ± 2 °C, pH 6.8–7.2 and continuous illumination) but in the absence of microorganisms. At 6, 12, 24, 36, 48, and 72 h, aliquots were centrifuged at 11,000× *g* for 40 min at 20 °C. The resulting supernatants were subsequently filtered through 3 kDa centrifugal ultrafiltration units (Sartorius, Göttingen, Germany) to separate ultrafilterable (dissolved) copper from particulate copper. Dissolved copper was quantified by flame atomic absorption spectrometry (FAAS) at 324.7 nm using certified copper standard solutions [28].

### 2.3. Nostoc linckia Strain and Cultivation Conditions

The cyanobacterium *Nostoc linckia* (Roth) Born. & Flah., strain CNMN-CB-03, was obtained from the National Collection of Non-Pathogenic Microorganisms of the Institute of Microbiology and Biotechnology, Technical University of Moldova.

The strain was cultivated in a mineral medium containing the following components (g/L): KNO_3_, 0.50; K_2_HPO_4_, 0.45; NaHCO_3_, 0.05; MgSO_4_·7H_2_O, 0.10; and CaCl_2_, 0.11; supplemented with the following trace elements (mg/L): ZnSO_4_·7H_2_O, 0.05; MnSO_4_, 2.00; H_3_BO_3_, 0.85; (NH_4_)_6_Mo_7_O_24_·4H_2_O, 2.25; FeSO_4_·7H_2_O, 4.00; Co(NO_3_)_2_·H_2_O, 0.009; and EDTA, 4.75. Cultivation was carried out in 100 mL flasks containing 50 mL of mineral medium adjusted to pH 6.8–7.2. Cultures were maintained at 25 ± 2 °C under continuous illumination (55 µmol photons m^−2^ s^−1^) and gently mixed at regular intervals to ensure homogeneous growth [14]. Copper compounds were added on the third day of cultivation, corresponding to the onset of the exponential growth phase. All experimental treatments received the same copper concentration, expressed as metallic copper equivalent (15 mg Cu/L), regardless of the chemical form applied (CuNPs, CuONPs, or CuSO_4_·5H_2_O). The concentration of 15 mg Cu L^−1^ was selected as a common copper-equivalent dose because it lies within the exposure interval defined by the previous experiments with CuNPs and CuONPs, in which measurable physiological and biochemical responses were observed while maintaining culture viability [29]. The control culture was grown under identical conditions without copper supplementation. The total cultivation period was 12 days.

### 2.4. Experimental Design

The experimental scheme consisted of three consecutive stages: (i) cultivation of *Nostoc linckia* in the presence of different copper forms, followed by evaluation of the quantity and biochemical composition of the produced biomass; (ii) characterization of the culture liquid; and (iii) evaluation of the effects of maize seed priming using the obtained culture liquid.

Four experimental treatments were established: control (without copper addition), CuSO_4_·5H_2_O (15 mg Cu/L), CuNPs (15 mg Cu/L), and CuONPs (15 mg Cu/L). Each treatment was performed with five independent biological replicates. At the end of the cultivation (the 12th day), the biomass production, photosynthetic pigments (total chlorophyll, carotenoids, and phycobiliproteins), macromolecular composition (proteins, carbohydrates, and lipids), antioxidant activity (ABTS and DPPH assays), lipid peroxidation level (malondialdehyde, MDA), and copper concentration in the culture liquid were determined. The culture liquid was subsequently characterized by its physicochemical and biochemical properties and used in the maize seed priming experiment.

### 2.5. Preparation and Biochemical Characterization of Nostoc linckia Biomass

At the end of the cultivation period, *Nostoc linckia* biomass was harvested by centrifugation at 5000× *g* for 10 min and washed once with deionized water. The biomass was subsequently resuspended in deionized water to a final concentration of 10 mg dry weight (DW)/mL and subjected to six consecutive freeze–thaw cycles (−20 °C/room temperature) to achieve cell lysis and the release of intracellular constituents.

Biomass concentration was determined spectrophotometrically based on a previously established calibration curve relating culture absorbance at 590 nm to dry biomass concentration [15,29].

Protein content in biomass was determined using the Lowry assay. A 0.1 mL aliquot of standardized biomass was mixed with 0.9 mL of 0.1 N NaOH and incubated at room temperature for 30 min. After alkaline hydrolysis, 0.2 mL of the hydrolysate was diluted with 0.8 mL of distilled water, followed by the addition of 1.5 mL of 49 mL of 2% sodium carbonate in 0.1 N NaOH mixed with 1 mL of a solution containing 0.5% CuSO_4_·5H_2_O and 1% sodium potassium tartrate. After incubation for 10 min at room temperature, 0.5 mL of 0.1 N Folin–Ciocalteu reagent was added, and the mixture was incubated in the dark for 40 min. Absorbance was measured at 750 nm against a reagent blank. Protein concentration was calculated from a calibration curve prepared using bovine serum albumin [15,30].

Carbohydrates were determined by the anthrone method. A 0.2 mL aliquot of the sample was mixed with 2.0 mL of anthrone reagent (0.5% anthrone in 66% sulfuric acid) and incubated in a boiling water bath for 10 min. After cooling, the samples were incubated at room temperature for 30 min, and absorbance was measured at 620 nm against a reagent blank. Carbohydrate concentration was calculated from a calibration curve prepared using glucose [15,31].

Total lipids were determined using the sulfo-phospho-vanillin method. Lipids were extracted from standardized biomass using a chloroform–ethanol mixture. The dried lipid extract was treated with 1.0 mL of concentrated sulfuric acid and heated at 100 °C for 30 min. After cooling, 3.0 mL of phospho-vanillin reagent (0.12 mg vanillin/mL in 68% phosphoric acid) was added, and the mixture was incubated in the dark at room temperature for 30 min. Absorbance was measured at 520 nm against a reagent blank. Lipid concentration was calculated from a calibration curve prepared using oleic acid [29,32].

Total chlorophyll and carotenoid contents were determined following extraction with 96% ethanol at room temperature for 12 h. After centrifugation, the absorbance of the extract was measured at 665, 649, and 450 nm. Chlorophyll a and carotenoid contents were calculated according to the equations of Lichtenthaler and expressed as a percentage relative to the dry biomass [33].

Phycobiliproteins were quantified according to Bennett and Bogorad. Standardized biomass (10 mg/mL), subjected to at least eight freeze–thaw cycles, was centrifuged at 11.000 rpm for 20 min, and the supernatant was used for analysis. Absorbance was measured at 620 and 650 nm for C-phycocyanin and allophycocyanin, respectively, and at 565 nm for R-phycoerythrin. Phycobiliprotein contents were calculated using the equations of Bennett and Bogorad (1973) [29,33,34].

Malondialdehyde (MDA) content, an indicator of lipid peroxidation, was determined using the thiobarbituric acid reactive substances (TBARS) assay. A total of 1.0 mL of the standardized biomass suspension was mixed with 2.0 mL of thiobarbituric acid reagent (0.67% thiobarbituric acid prepared in 20% trichloracetic acid). The mixture was incubated in a boiling water bath (100 °C) for 20 min, rapidly cooled, and centrifuged. The absorbance of the supernatant was measured at 532 nm, with nonspecific absorbance corrected at 600 nm. MDA content was calculated using the extinction coefficient of the MDA–TBA complex and expressed as nmol g^−1^ dry weight [23].

The antioxidant activity of the biomass extracts was evaluated using the ABTS [35] and DPPH [36] radical scavenging assays. Antioxidant activity was determined using the ABTS [2,2′-azinobis(3-ethylbenzothiazoline-6-sulfonic acid)] assay, which is based on the reduction of the ABTS radical cation by antioxidants present in the sample. The ABTS radical was generated by reacting a 7 mM ABTS solution with 2.45 mM potassium persulfate and incubating the mixture in the dark for 12–16 h. Before use, the working solution was adjusted to an absorbance of 0.70 ± 0.02 at 734 nm. The reaction mixture consisted of 0.3 mL of sample and 2.7 mL of the ABTS working solution and was incubated at room temperature for 6 min. Absorbance was measured at 734 nm, and antioxidant activity was expressed as the percentage inhibition of the ABTS radical.

Antioxidant activity, determined using the DPPH (2,2-diphenyl-1-picrylhydrazyl) assay, is based on the reduction of the stable DPPH radical by antioxidants present in the sample [36]. The DPPH working solution (0.05 mM) was prepared in 96% ethanol and adjusted to an absorbance of 0.64–0.66 at 517 nm before use. The reaction mixture consisted of 0.3 mL of sample and 2.7 mL of DPPH solution and was incubated in the dark at room temperature for 30 min. Absorbance was measured at 517 nm, and antioxidant activity was expressed as the percentage inhibition of the DPPH radical.

### 2.6. Preparation and Characterization of the Culture Liquid

Following biomass separation, the entire volume of the cell-free culture liquid was freeze-dried. Freeze-drying was applied to concentrate the culture liquid prior to biochemical analyses. The resulting dry residue was stored at −20 °C and reconstituted in deionized water immediately before biochemical analyses to obtain a standardized dry matter concentration of 1 mg mL^−1^ for analysis and 0.1 mg L^−1^ for priming, ensuring comparable conditions for all subsequent analyses.

The freeze-dried culture liquid, reconstituted in deionized water, was characterized by its physicochemical and biochemical properties. Protein and carbohydrate contents, as well as antioxidant activity (ABTS), were determined using the same analytical procedures applied to cyanobacterial biomass.

Fourier transform infrared (FTIR) spectroscopy was performed directly on the freeze-dried samples without further pretreatment. Spectra were recorded over the range of 4000–400 cm^−1^ using a PerkinElmer FTIR spectrometer (PerkinElmer Inc., Waltham, MA, USA) equipped with an air-cooled DTGS detector.

The pH and electrical conductivity of the reconstituted culture liquid were measured at 20 ± 0.5 °C using a laboratory a MultiLine^®^ Multi 3630 IDS multiparameter meter (WTW, Weilheim, Germany) equipped with appropriate electrodes, calibrated with standard buffer solutions. Copper concentration in the culture liquid was quantified by flame atomic absorption spectrometry (FAAS) at 324.7 nm using certified copper standard solutions [28].

### 2.7. Seed Priming Experiment and Germination Assessment

Seeds of four maize (*Zea mays* L.) hybrids (374, 310, 458, and 461) were obtained from the National Center for Seed Research and Production (Republic of Moldova).

Seeds were surface sterilized by immersion in 70% ethanol for 3 min, followed by immersion in 1% sodium hypochlorite for 3 min, and then rinsed three times with sterile deionized water.

Seed priming was performed by soaking the seeds for 24 h in solutions prepared by reconstituting the freeze-dried liquid filtrates to a final concentration of 0.1 mg dry matter/mL. Control seeds were incubated in deionized water under identical conditions.

Following priming, seeds were evenly distributed in Petri dishes lined with moistened filter paper and incubated at 25 ± 1 °C in the dark. Each treatment consisted of five independent biological replicates, each with 20 seeds.

Seed germination was monitored every 12 h, and the number of normally germinated seedlings was recorded in accordance with the International Seed Testing Association (ISTA) rules. Representative photographs documenting the seed germination experiment at different experimental stages are provided in the Supplementary Materials (Figure S1).

#### Germination Parameters

The following germination parameters were calculated:

Germination Percentage (GP):

GP (%) = Ng/Nt × 100(1)

where Ng is the number of normally germinated seeds, and Nt is the total number of tested seeds.

Mean Germination Time (MGT):

MGT (h) = ∑(*ni* × *ti*)/∑*ni*, (2)

where *ni* is the number of newly germinated seeds, and *ti* is the time (h) elapsed from the beginning of the germination test to the *i*th time point.

Germination Index (GI):

GI = ∑Gt/Tt,(3)

where Gt is the number of seeds germinated at time t, and Tt is the corresponding time elapsed from the beginning of the experiment.

Based on germination percentage and seedling growth parameters, the following vigor indices were calculated:

SVI-I = GP × L(4)

SVI-II = GP × DW(5)

where L is the mean seedling length (cm) and DW is the mean seedling dry weight (g).

### 2.8. Biochemical Characterization of Maize Seedlings

Seedlings were harvested on the fourth day after germination. Fresh weight and seedling length were measured immediately after harvesting. Samples were subsequently dried to constant weight and analyzed for total chlorophyll and carotenoid contents, total phenolic compounds [37], lipid peroxidation (MDA), and antioxidant activity using the ABTS and DPPH assays.

The biochemical analyses were performed using the same methods as for *Nostoc linckia* biomass.

### 2.9. Statistical Analysis

All experiments were performed using five independent biological replicates. Results are presented as the mean ± standard deviation (SD).

Biomass and culture liquid data were analyzed using one-way analysis of variance (one-way ANOVA), followed by Tukey’s honestly significant difference (HSD) post hoc test. Seed germination, seedling growth, and biochemical parameters were analyzed using two-way analysis of variance (two-way ANOVA), considering maize hybrid and seed priming treatment as fixed factors, followed by Tukey’s HSD multiple comparison test. Detailed results of the one-way and two-way ANOVA analyses, including F-values, degrees of freedom (df), and exact *p*-values, are provided in the Supplementary Materials (Tables S1 and S2).

Pearson correlation analysis, hierarchical clustering heatmap analysis, and principal component analysis (PCA) were performed to investigate the relationships among cyanobacterial physiological traits, culture filtrate characteristics, and maize responses.

Differences were considered statistically significant at *p* < 0.05. All statistical analyses were performed using SPSS Statistics version 19.0.

## 3. Results

### 3.1. Release of Dissolved Copper from CuNPs and CuONPs

The release of dissolved copper from CuNPs and CuONPs was evaluated in sterile mineral medium under the same physicochemical conditions as those used in the biological experiments (Table 1).

Distinct dissolution profiles were observed for the two types of nanoparticles. CuNPs exhibited a gradual increase in the concentration of dissolved copper during the first 24 h of incubation, followed by stabilization with only minor changes thereafter. In contrast, CuONPs released higher amounts of dissolved copper during the initial incubation period (6–12 h), after which the dissolved copper concentration gradually decreased.

Despite these differences in dissolution kinetics, the concentration of dissolved copper remained low throughout the incubation period for both types of nanoparticles, indicating that only a small fraction of the total copper was converted into dissolved species under the experimental conditions. Consequently, both nanoparticle suspensions remained predominantly in particulate form throughout the exposure period.

### 3.2. Effects of Different Copper Forms on the Growth and Biochemical Composition of Nostoc linckia Biomass

Exposure of *Nostoc linckia* cultures to different copper forms elicited distinct physiological and metabolic responses, demonstrating that the chemical form of copper influences both biomass accumulation and the distribution of the major metabolite classes (Table 2).

The most pronounced differences were observed in biomass accumulation. Treatment with CuNPs and CuSO_4_ significantly reduced the final biomass by approximately 24% and 27%, respectively, compared with the control (*p* < 0.05), with no significant difference between the two treatments. In contrast, exposure to CuONPs stimulated culture growth, resulting in a final biomass approximately 15% higher than that of the control and significantly greater than in all other experimental variants (*p* < 0.05).

The growth responses were accompanied by marked changes in primary metabolism. The greatest reduction in protein content was observed in cultures treated with CuNPs and CuSO_4_, where the protein levels decreased by 40–45% relative to the control (*p* < 0.05). The CuSO_4_ treatment exhibited the lowest protein content, significantly lower than all other experimental variants, whereas no significant differences from the control were detected in the biomass of the CuONP-treated cultures. In contrast to proteins, the total carbohydrate content remained essentially unchanged in cultures exposed to both nanoparticle treatments, whereas exposure to CuSO_4_ resulted in a significant increase of approximately 14% compared with the control.

Photosynthetic pigments responded differently to the three copper forms. The strongest inhibition of chlorophyll content was observed in the CuSO_4_ treatment, where chlorophyll levels were approximately 34% lower than in the control (*p* < 0.05). In contrast, CuONPs significantly increased the chlorophyll content, whereas CuNPs caused only a moderate reduction. An even more pronounced response was observed for phycobiliproteins, whose content decreased more than fourfold in both the CuNP and CuSO_4_ treatments. In the CuONP treatment, phycobiliprotein content remained at approximately 86% of the control level and was significantly higher than in the other two copper-treated variants.

Lipid metabolism exhibited a response distinct from that of proteins and photosynthetic pigments. Both types of nanoparticles significantly increased the lipid content by approximately 77–79% compared with the control (*p* < 0.05), with no significant differences between the CuNP and CuONP treatments. In contrast, CuSO_4_ induced only a slight increase in lipid accumulation. The increase in lipid content was accompanied by enhanced lipid peroxidation, as indicated by elevated malondialdehyde (MDA) levels. The highest MDA concentrations were observed in the CuNP and CuONP treatments, which exceeded the control values by more than twofold (*p* < 0.05), whereas the CuSO_4_·5H_2_O treatment resulted in only a moderate increase.

Evaluation of antioxidant activity revealed marked differences between the two analytical methods employed. The DPPH radical-scavenging activity was less affected by the treatments, with the highest value observed in the CuONP treatment, followed by the control, whereas the CuNP and CuSO_4_ treatments exhibited lower activities. In contrast, antioxidant activity, as determined by the ABTS assay, was considerably more sensitive to copper exposure. Relative to the control, ABTS radical-scavenging activity decreased by approximately 47% in the CuNP treatment, 34% in the CuONP treatment, and 52% in the CuSO_4_ treatment, with all differences being statistically significant (*p* < 0.05).

### 3.3. Effects of Different Copper Forms on the Biochemical Composition and Physicochemical Properties of Culture Liquid

**Figure 1:**
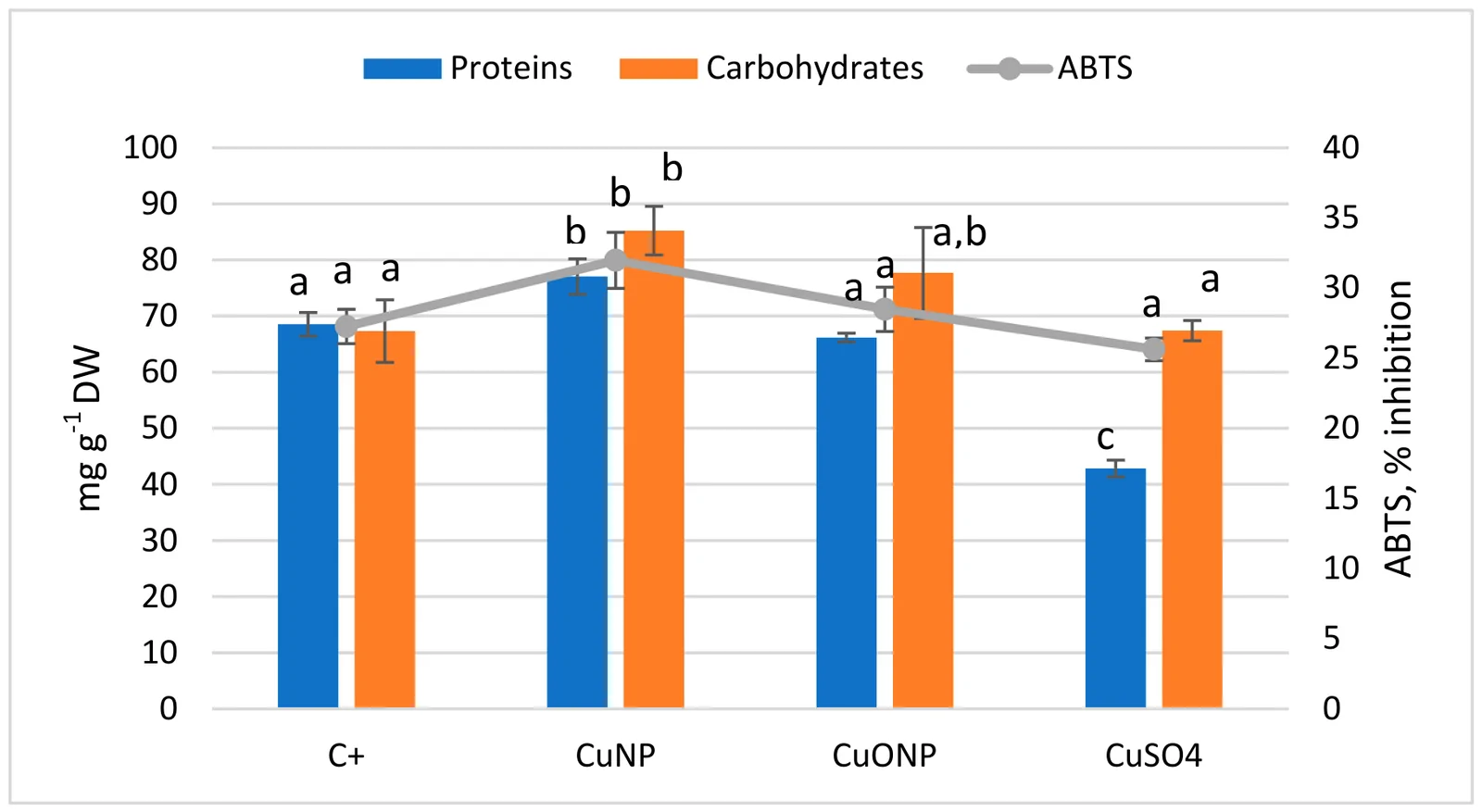
Biochemical composition and antioxidant activity of the culture liquid obtained from *Nostoc linckia* cultivated in the presence of different copper forms. Data are presented as mean ± SD (*n* = 5). Columns or points sharing different superscript letters differ significantly according to one-way ANOVA followed by Tukey’s HSD post hoc test (*p* < 0.05).

Analysis of the culture liquid revealed that the chemical form of copper significantly influenced the biochemical composition of extracellular metabolites released by *Nostoc linckia* (Figure 1).

The extracellular protein content varied significantly among the experimental treatments. The highest concentration was observed in the culture liquid from the CuNP-treated cultures, exceeding that of the control by approximately 14% (*p* < 0.05). In contrast, the CuSO_4_ treatment resulted in the lowest protein concentration, which was significantly lower than that of all the other experimental variants. In the CuONP treatment, extracellular protein content did not differ significantly from the control.

Extracellular carbohydrate content was also affected by the treatments. The highest values were observed in the CuNP and CuONP treatments; however, only the increase induced by CuNPs was significantly higher than that of the control and the CuSO_4_ treatment. The CuONP treatment showed intermediate values that did not differ significantly from those of the other experimental variants.

The antioxidant activity of the culture liquid, determined using the ABTS assay, followed a pattern like that observed for extracellular protein content. The highest ABTS radical-scavenging activity was detected in the CuNP treatment, where it was significantly greater than in all other experimental variants (*p* < 0.05). In contrast, the CuONP, CuSO_4_, and control treatments did not differ significantly from one another (Figure 1).

FTIR spectroscopy demonstrated that changes in the biochemical composition were accompanied by changes in the culture liquid (Figures S2 and S3). All treatments exhibited characteristic absorption bands of the major classes of organic compounds; however, their intensities and spectral profiles varied depending on the chemical form of copper used during cultivation. The most evident variations occurred in the regions corresponding to hydroxyl and amino groups (3200–3400 cm^−1^) [38], carbonyl and amide I groups (1700–1600 cm^−1^) [39], and the carbohydrate- and polysaccharide-associated region (1200–900 cm^−1^) [38,39]. Compared with the control, culture liquid obtained from the CuNP- and CuONP-treated cultures exhibited more evident variations in the bands attributed to protein and carbohydrate fractions, whereas the CuSO_4_ treatment was distinguished by specific changes in the 1270–1240 cm^−1^ region, assigned to oxygen-containing functional groups (C–O, P=O, and S=O) [38,40]. Overall, the FTIR spectra suggest that the different copper forms did not induce the production of new classes of extracellular metabolites but rather modified the spectral profile of the major organic compounds secreted by *Nostoc linckia*.

The physicochemical properties of the culture liquid were less affected by the treatments (Table 3). Compared with the control, nanoparticle treatments resulted in a slight increase in pH, with the highest value observed with the CuONP treatment, whereas the CuSO_4_ treatment decreased the medium pH. Electrical conductivity remained essentially unchanged in the nanoparticle-treated variants and showed only a moderate reduction in the CuSO_4_ treatment.

Compared with the control, nanoparticle treatments resulted in a slight increase in culture liquid pH, from 9.15 to 9.38 with CuNP treatment and to 9.63 with CuONP treatment, whereas exposure to CuSO_4_ reduced the pH to 8.65. Electrical conductivity remained virtually unchanged in the nanoparticle-treated variants (1620–1621 μS cm^−1^) but decreased in the CuSO_4_ treatment, reaching 1528 μS cm^−1^.

Determination of the residual copper concentration at the end of the cultivation period revealed marked differences among the three copper forms. The lowest residual copper concentration in the culture liquid was detected in the CuNP treatment (0.16 ± 0.04 mg/L), followed by the CuONP treatment (0.96 ± 0.10 mg/L), whereas the highest residual concentration was recorded in the CuSO_4_ treatment (1.64 ± 0.27 mg/L). In the control culture liquid, copper concentration was below the detection limit.

### 3.4. Effect of the Culture Liquid on Seed Germination, Seedling Growth, and Vigor Parameters of Maize

To evaluate the biostimulatory activity of the culture liquid, four locally developed maize hybrids (374, 310, 458, and 461), representing genotypes with different physiological responses to seed priming treatments, were used. Seeds were treated with deionized water (C−) as the negative control, culture liquid obtained from *Nostoc linckia* grown under standard conditions (C+) as the positive control, or culture liquid collected from cultures exposed to CuNPs, CuONPs, and CuSO_4_ to assess their effects. These effects were then evaluated by measuring the germination percentage (GP), mean germination time (MGT), and germination index (GI) (Table 4).

Germination percentage (GP) remained high across all four maize hybrids, ranging from 85% to 100%, with no statistically significant differences among treatments within each hybrid (*p* > 0.05). These results indicate that the seed priming treatments did not affect final germination capacity, regardless of genotype.

In contrast to GP, mean germination time (MGT) was more sensitive to the treatments, revealing hybrid-dependent responses. The most pronounced changes were observed in hybrid 374, where culture liquid obtained from the CuONP- and CuNP-treated *Nostoc linckia* cultures significantly reduced the germination time compared with the negative control and the CuSO_4_ treatment. In hybrid 461, a clear reduction in MGT was observed following the CuONP treatment, whereas in hybrid 458, the differences among treatments were less pronounced, with a significant delay in germination occurring only in the CuSO_4_ treatment. Hybrid 310 showed no significant differences among treatments.

The germination index (GI), which integrates both germination percentage and germination rate, proved to be the most sensitive indicator of the biostimulatory effect. In hybrid 374, the highest GI values were observed in the CuNP and CuONP treatments, which were significantly higher than those in the control and CuSO_4_ treatments. A similar, although less pronounced, response was observed in hybrid 461, where the CuONP treatment produced the highest germination index. Hybrid 310 exhibited no significant differences among treatments, whereas in hybrid 458, only the CuSO_4_ treatment significantly reduced GI compared with the positive control.

Overall, these results demonstrate that the effect of the culture liquid on germination depends on both the chemical form of copper used during *Nostoc linckia* cultivation and the maize hybrid genotype. Although the final germination percentage was only marginally affected by the treatments, the parameters describing germination dynamics, particularly MGT and GI, clearly distinguished the experimental variants.

The effects of the seed priming treatments on early seedling development were evaluated by determining the total seedling length, fresh weight, dry weight, and the seedling vigor indices SVI-I and SVI-II (Table 5). These seedling metrics complemented the germination parameters and showed much stronger genotype-dependent responses, with significant differences observed both among hybrids and among treatments.

Seedling length exhibited a genotype-dependent response. In hybrid 374, the greatest seedling length was recorded in seeds primed with culture liquid obtained from *Nostoc linckia* cultures treated with CuSO_4_, whereas in hybrid 458, seedling growth was significantly stimulated by the CuONP treatment as well as by the culture liquid from the untreated *Nostoc linckia* culture (C+). No statistically significant differences among treatments were observed for hybrids 310 and 461.

More pronounced effects were observed on biomass accumulation. In hybrid 310, seeds primed with culture liquid from CuSO_4_-treated *Nostoc linckia* cultures exhibited the highest fresh and dry biomass, with values significantly exceeding those of all other treatments. A similar, although less pronounced, response was observed in hybrid 458. In hybrid 461, the greatest biomass accumulation was observed following priming with culture liquid from the untreated *Nostoc linckia* culture (C+), whereas in hybrid 374, the treatments did not significantly affect seedling biomass.

The seedling vigor index SVI-I showed only minor variation among treatments, with significant differences detected only in hybrid 458. In contrast, SVI-II proved to be the most sensitive indicator of seed priming response. The highest SVI-II values were recorded in hybrids 310 and 458 following priming with culture liquid obtained from CuONP-treated *Nostoc linckia* cultures, whereas in hybrid 461, the maximum value was observed in the C+ treatment. No significant differences among treatments were detected for hybrid 374.

Overall, the results indicate that the response to seed priming depended on both the maize hybrid genotype and the biological properties of the culture liquid produced under the different cultivation conditions of *Nostoc linckia*. Although the final germination percentage was only marginally affected, the parameters describing germination dynamics, particularly mean germination time (MGT) and germination index (GI), clearly differentiated among treatments. In contrast, the parameters associated with early seedling growth and vigor exhibited stronger genotype-dependent responses, indicating that the biostimulatory potential of the culture liquid was influenced by both the copper form used during *Nostoc linckia* cultivation and the genetic background of the maize hybrids.

### 3.5. Effect of Seed Priming on Biochemical Status of Maize Seedlings

**Figure 2:**
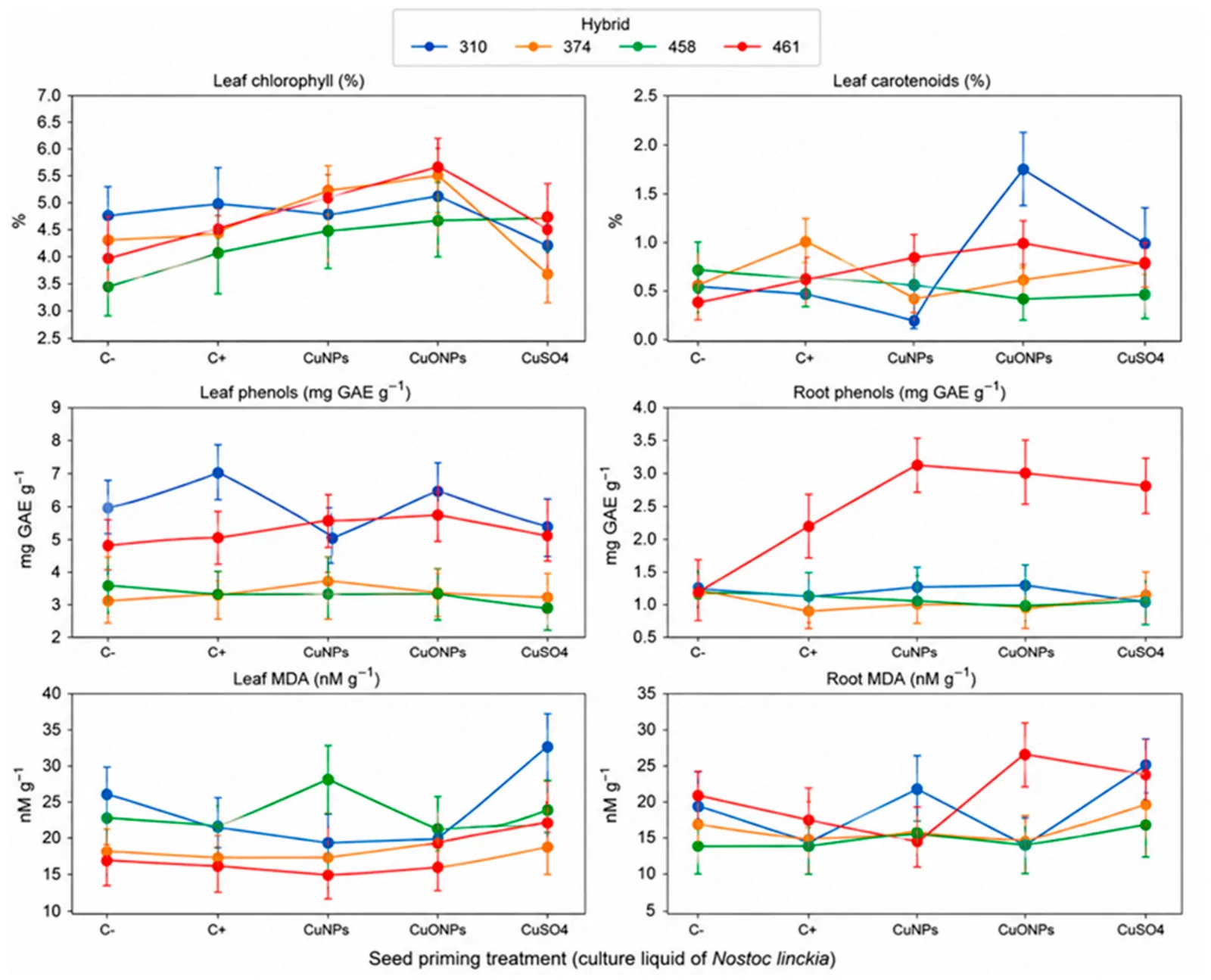
Biochemical responses of maize seedlings to seed priming with Nostoc linckia culture liquid obtained under different copper treatments. Treatment: C−, deionized water; C+, culture liquid from control Nostoc; CuNPs, CuONPs, CuSO_4_—culture liquids from Nostoc grown with corresponding copper forms; Data are presented as mean ± SD (n = 5). Complete statistical analysis, including mean values, standard deviations and Tukey’s HSD multiple comparison test, is provided in Table S1.

To evaluate whether the changes induced in the composition of the *Nostoc linckia* culture liquid were reflected in plant metabolism, photosynthetic pigments in the leaves and oxidative stress markers in the leaves and roots of maize seedlings were determined. The complete biochemical dataset, together with the statistical analyses, is presented in Table S3, whereas the most representative parameters are shown in Figure 2.

Overall, the biochemical responses of maize seedlings depended on both the seed genotype (hybrid) and the culture liquid used for seed priming. The most pronounced effects were observed in seedlings originating from seeds primed with culture liquid obtained from *Nostoc linckia* cultivated in the presence of CuONPs.

Biochemical responses differed among maize hybrids, treatments, plant organs, and the parameters analyzed. In hybrid 310, seed priming with culture liquid obtained from the CuNP-treated culture significantly reduced the leaf ABTS and DPPH radical-scavenging activities and phenolic content compared with both control treatments. Similar reductions were observed following priming with the CuSO_4_-derived culture liquid, whereas the CuONP-derived culture liquid did not significantly affect these parameters. Leaf MDA content was significantly lower after priming with CuNP- and CuONP-derived culture liquids than in both control treatments, while the CuSO_4_-derived culture liquid resulted in the highest MDA level. Chlorophyll content remained at the control level after priming with the CuNP- and CuONP-derived culture liquids but decreased significantly following treatment with the CuSO_4_-derived culture liquid. Carotenoid content exhibited a distinct response, decreasing significantly in all copper treatments, with the greatest reductions observed after priming with CuNP- and CuSO_4_-derived culture liquids. In roots, ABTS and DPPH activities and phenolic content did not differ significantly among treatments. Root MDA content was lowest in the CuONP treatment, whereas the CuNP and CuSO_4_ treatments maintained MDA levels comparable to the controls.

In hybrid 374, leaf ABTS activity did not differ significantly among the CuNP-, CuONP-, CuSO_4_-derived culture liquids, and the water control. No significant treatment-related differences were detected in leaf DPPH activity, phenolic content, or MDA. Chlorophyll content was significantly lower after priming with the CuSO_4_-derived culture fluid than in all other treatments. Carotenoid content decreased in all copper treatments compared with the control. None of the measured root parameters differed significantly among treatments.

In hybrid 458, leaf ABTS and DPPH activities, phenolic content, chlorophyll, and carotenoid contents did not differ significantly among treatments. Leaf MDA content was significantly lower in the CuONP treatment than in the other treatments. No statistically significant differences were observed among treatments in root ABTS and DPPH activities, phenolic content, or MDA.

In hybrid 461, no significant treatment-related differences were detected in leaf ABTS and DPPH activities, phenolic content, or MDA. Leaf chlorophyll content was significantly reduced only after priming with the CuONP-derived culture liquid, whereas carotenoid content was significantly higher in all treatments with Nostoc culture liquids than in the water control. In roots, ABTS activity was significantly higher in all treatments with Nostoc culture liquids except CuSO_4_, where values remained at the control level. The same response pattern was observed for DPPH activity. Root phenolic content was significantly higher after priming with all Nostoc culture liquids than in the water control, while root MDA content was significantly lower in the CuNP and CuONP treatments than in the control.

Overall, the biochemical responses to seed priming varied markedly among maize hybrids and depended on the analyzed parameter and plant organ. Hybrid 310 exhibited the largest number of significant changes in leaf biochemical traits. In hybrid 374, significant treatment effects were confined to photosynthetic pigments, including reduced chlorophyll content after priming with the CuSO_4_-derived culture liquid and lower carotenoid levels in all copper treatments. In hybrid 458, treatment effects were restricted primarily to leaf MDA, whereas hybrid 461 responded predominantly through changes in photosynthetic pigments in leaves and antioxidant-related parameters in roots.

The observed biochemical changes were consistent with the previously observed improvements in seedling growth and vigor, suggesting that the copper-induced modifications in the composition of the *Nostoc linckia* culture liquid influenced not only the germination performance, but also the metabolic activity of the seedlings.

Changes in photosynthetic pigments were accompanied by coordinated changes in oxidative stress biomarkers. Treatments that promoted higher chlorophyll accumulation also tended to maintain lower malondialdehyde (MDA) levels and higher phenolic compound contents, indicating an improved physiological status of the seedlings. Although the magnitude of these responses varied among hybrids, similar trends were observed across most experimental treatments.

## 4. Discussion

The present study demonstrates that the biological activity of *Nostoc linckia* is governed not only by the copper concentration, but more importantly, by copper form. Although all treatments had the same total copper concentration, CuNPs, CuONPs, and CuSO_4_ induced responses that propagated from cyanobacterial physiology to culture liquid composition, and ultimately to maize seedling performance. This integrated response indicates that the culture liquid reflects the metabolic status of the producing organism and can therefore serve as a sensitive indicator of elicitor-induced modifications. The mechanisms underlying these responses can be interpreted considering the physicochemical properties of the different copper forms and their interactions with cyanobacterial cells. These interactions may induce metabolic remodeling, which can be reflected in changes in the composition of the culture liquid and its biological activity.

The two nanoparticle types exhibited distinct dissolution profiles, with CuONPs releasing higher amounts of dissolved copper during the first 12 h, whereas CuNPs showed a gradual increase followed by stabilization. Nevertheless, in both cases, only a small fraction of the total copper was converted into dissolved species, indicating that the suspensions remained predominantly particulate throughout the exposure period. These findings are consistent with previous reports demonstrating that the biological effects of Cu- and CuO-based nanoparticles depend not only on dissolved copper, but also on nanoparticle-specific properties, including dissolution behavior and particle-related interactions [41]. Therefore, the contrasting responses of *Nostoc linckia* to CuNPs and CuONPs are unlikely to be explained solely by dissolved copper release. The distinct behavior of cultures treated with CuONPs deserves particular attention. Unlike CuNPs and CuSO_4_, copper oxide nanoparticles stimulated biomass accumulation while maintaining protein, chlorophyll, and phycobiliprotein levels close to those of the control, despite a significant increase in lipid and malondialdehyde (MDA) contents. The simultaneous increase in biomass and MDA seems contradictory, as MDA is commonly considered an indicator of oxidative damage. This response suggests that the oxidative processes induced by CuONPs occurred concomitantly with an efficient metabolic adaptation. The increased lipid content indicates an enhanced biosynthesis and remodeling of membrane lipids, processes that inevitably increase the amount of lipid substrate available for peroxidation. Consequently, the increased MDA levels may also reflect an increase in lipid turnover. Lately, it has been increasingly stated that MDA, depending on the physiological context and the efficiency of redox regulation systems, can be considered not only as a marker of lipid peroxidation, but also signaling molecules involved in stress acclimation and the activation of defense responses, and the change in its content should be analyzed in the context of changes in other metabolic parameters [23,42]. Such a response as that of *Nostoc linckia* to the action of CuONPs is characteristic of hormesis and aligns with the concept of metabolic elicitation introduced earlier, whereby moderate stress promotes metabolites with enhanced functional value. Similar responses have been reported in cyanobacteria and microalgae exposed to metallic nanoparticles, where moderate oxidative stress sustained growth and drove metabolic remodeling, with the magnitude of the effects depending on nanoparticle characteristics and stress intensity [22,23,43,44]. However, the proposed interpretation in terms of hormesis and metabolic adaptation is based on the integrated physiological and biochemical responses observed in this study and should not be regarded as direct evidence of the underlying mechanisms. Elucidation of the signaling pathways involved would require complementary analyses of antioxidant enzyme activities, non-enzymatic antioxidants, osmoprotectants, and stress-related gene expression, which were beyond the scope of the present work.

In contrast, CuNPs caused the most pronounced reductions in protein and phycobiliprotein contents, accompanied by increased lipid accumulation and elevated MDA levels. This metabolic profile indicates that metabolic fluxes were redirected from protein biosynthesis and photosynthetic activity toward stress protection and adaptation mechanisms. The marked decline in phycobiliproteins is particularly significant because these pigments constitute the principal light-harvesting complexes in cyanobacteria and are among the most sensitive indicators of impairment of the photosynthetic apparatus. Our findings are consistent with those reported by Pandey et al., who demonstrated that exposure to elevated concentrations of copper ions and copper nanoparticles decreases photosynthetic pigment content while intensifying oxidative stress in cyanobacteria [22].

The ionic form of copper elicited a response distinct from that induced by the nanoparticles, characterized by reduced biomass, protein content, and photosynthetic pigments, accompanied by increased carbohydrate accumulation. This metabolic pattern suggests that the predominant effect of CuSO_4_ was the suppression of growth and biosynthetic processes, accompanied by a redistribution of carbon flux toward carbohydrate accumulation. This response contrasts sharply with the profile observed in the CuONP treatment, suggesting that the immediate bioavailability of copper ions preferentially inhibits biosynthetic processes, whereas the gradual release of copper from CuONPs allows for the development of an adaptive metabolic response. Similar responses have been reported in photosynthetic microorganisms exposed to various environmental stressors, in which carbohydrate accumulation has been interpreted as a mechanism for carbon conservation and metabolic adaptation [45]. Moreover, different external factors may modify not only the total carbohydrate content, but also the relative proportions of carbohydrate fractions in cyanobacterial biomass [46]. Furthermore, comparative studies in cyanobacteria have shown that the ionic form of copper exerts a stronger inhibitory effect on growth and the photosynthetic apparatus than certain nanoparticulate forms, confirming that copper’s chemical form is a major determinant of the physiological response [22].

An important finding of the present study is the difference between the two methods used to evaluate antioxidant capacity. The greater reduction in antioxidant capacity measured by the ABTS assay compared with the DPPH assay indicates that these assays responded differently to the biochemical changes induced by copper exposure. Because ABTS and DPPH assess the overall in vitro antioxidant capacity of complex biological extracts and differ in their reaction mechanisms, the observed differences should not be interpreted as evidence for distinct fractions of the cellular antioxidant system. Rather, they suggest changes in the overall antioxidant composition of the extracts. Identification of the specific antioxidant molecules responsible for these responses would require targeted biochemical analyses beyond the scope of the present study. This observation supports recent recommendations advocating the complementary use of multiple antioxidant assays to more comprehensively characterize the antioxidant capacity of algal and cyanobacterial biomass [47,48].

Overall, our results support the hypothesis that different chemical forms of copper induce not only quantitative differences in growth, but also distinct metabolic strategies in *Nostoc linckia*. Among the tested copper forms, CuONPs provided the most balanced overall metabolic response, maintaining cyanobacterial growth while promoting favorable biochemical changes associated with enhanced biostimulatory activity.

The chemical form of copper influences not only the biomass composition of *Nostoc linckia*, but also the characteristics of the culture liquid. Although cyanobacterial biomass has traditionally been regarded as the primary biotechnological product, recent studies have demonstrated that the culture medium contains a complex mixture of proteins, extracellular polysaccharides (EPSs), amino acids, organic acids, phenolic compounds, and signaling molecules that can independently exert biological effects on plants and microorganisms or contribute to the survival and persistence of cyanobacterial cultures [49,50]. This observation is particularly relevant to the circular bioeconomy, as the culture liquid is still often regarded as a by-product of cyanobacterial cultivation, despite its potential as a valuable source of bioactive compounds.

In the present study, the CuNP treatment resulted in the greatest accumulation of extracellular proteins, carbohydrates, and higher antioxidant capacity, whereas CuSO_4_ produced the opposite effect. Numerous studies have demonstrated that the extracellular matrix of cyanobacteria has a high capacity to bind metal cations due to the abundance of carboxyl, hydroxyl, phosphate, and sulfate functional groups in extracellular polysaccharides and glycoproteins [51]. However, the metal-binding capacity of the extracellular matrix is not the only mechanism explaining the reduced amount of residual copper. Copper can also be eliminated by other mechanisms, including increased cellular uptake and the internalization of copper nanoparticles. The present study does not allow us to distinguish between these mechanisms. The FTIR analysis further supports these observations. Although all treatments exhibited the same general classes of functional groups, changes in the intensity of the absorption bands corresponding to proteins and polysaccharides indicate remodeling of the molecular composition of the extracellular fraction. The absence of new absorption bands, together with the predominant changes in the intensity of existing bands, indicates that copper treatments did not induce the synthesis of new classes of extracellular metabolites but rather altered the relative proportions of the major components of the culture liquid. This type of response is characteristic of stress-induced modifications and has previously been described for cyanobacterial EPSs produced under changing environmental conditions [49].

The residual copper measurements complement this interpretation. After 12 days of cultivation, the lowest amount of copper remained in the culture liquid of the CuNP-treated cultures, whereas progressively higher residual concentrations were detected in the CuONP and CuSO_4_ treatments. These differences indicate that copper bioavailability is strongly dependent on its chemical form. Studies on marine microalgae have likewise shown that copper and copper oxide nanoparticles undergo distinct transformation and ion-release processes, resulting in biological responses that differ from those elicited by ionic copper [52].

Taken together, these findings demonstrate that the culture liquid is a dynamic biological product whose composition is dependent of the chemical form of copper applied during cultivation. This observation has important implications for the development of cyanobacteria-based biostimulants, as it suggests that optimizing cultivation conditions can be used not only to modify biomass quality, but also to tailor the functional properties of culture liquid.

As one of the world’s major staple crops, maize plays a crucial role in ensuring global food security and therefore remains a major focus of agricultural research. In this context, the application of biostimulants, including those derived from cyanobacteria, at different stages of plant development, has emerged as a promising strategy for improving crop performance [53]. Seed priming with various types of biostimulants has attracted increasing attention because it can provide multiple agronomic benefits, ranging from enhanced germination and improved seedling vigor to increased productivity and greater tolerance to a variety of abiotic stresses, including heavy metal toxicity [54]. Our results demonstrate that the culture liquid of *Nostoc linckia* primarily influences germination dynamics and the early development of maize seedlings. The effects were evaluated using four commercial maize hybrids and were more pronounced in seedling vigor than in final germination capacity. In the present study, germination percentage (GP) remained high and was not significantly affected by the treatments, whereas mean germination time (MGT), germination index (GI), and particularly, seedling vigor parameters responded to the treatment. These findings are consistent with previous reports on cyanobacteria-derived biostimulants, which primarily enhance the rate of physiological processes and subsequent seedling development rather than altering the final germination percentage [55].

Hybrids 310 and 458 responded most favorably to priming with culture liquid obtained from CuONP-treated *Nostoc linckia* cultures, exhibiting significant increases in fresh weight, dry weight, and the SVI-II vigor index. In contrast, hybrid 374 showed only minor changes in biomass accumulation, whereas in hybrid 461, the greatest response was observed following treatment with culture liquid from the untreated *Nostoc linckia* culture (C+). The observed genotype-dependent responses indicate that the biological activity of the culture liquid is influenced by the genetic background of the maize hybrids. Although the molecular basis of this variability was not investigated, it likely reflects inherent differences in the perception and physiological response to the complex mixture of bioactive compounds present in the culture liquid. Elucidation of the signaling pathways and regulatory networks responsible for these genotype-specific responses requires dedicated molecular studies and was beyond the scope of the present work.

In the present study, treatments that promoted biomass accumulation and increased seedling vigor indices were generally associated with higher chlorophyll and phenolic compound contents in seedlings, together with reduced malondialdehyde (MDA) accumulation, indicating an improved physiological status of the seedlings.

Our findings are also consistent with experimental studies on maize seed priming, in which biostimulant treatments improved chlorophyll content, reduced MDA accumulation, enhanced antioxidant capacity, and promoted seedling biomass production. For example, Li and co-workers demonstrated that biopriming maize seeds with *Bacillus* cultures reduced lipid peroxidation and improved seedling physiological status by activating antioxidant defense mechanisms, with the magnitude of the response depending on both genotype and treatment [56].

The variability observed among the four maize hybrids further confirms that the efficacy of microbial biostimulants largely depends on the host plant’s genetic background. This observation is consistent with recent reviews of microalgae- and cyanobacteria-based biostimulants, which emphasize that physiological and biochemical responses can vary considerably among plant species and genotypes, even under similar experimental conditions [5]. In addition, in our study, residual copper present in the priming solutions should be considered when interpreting the observed plant responses. Based on the measured residual copper content in the culture liquids and the reconstitution procedure, the calculated copper concentrations in the priming solutions were 0.050 ± 0.006, 0.331 ± 0.014, and 0.547 ± 0.009 mg L^−1^ for the CuNP-, CuONP-, and CuSO_4_-derived culture liquids, respectively. Thus, a contribution of residual copper to the observed effects cannot be excluded.

**Figure 3:**
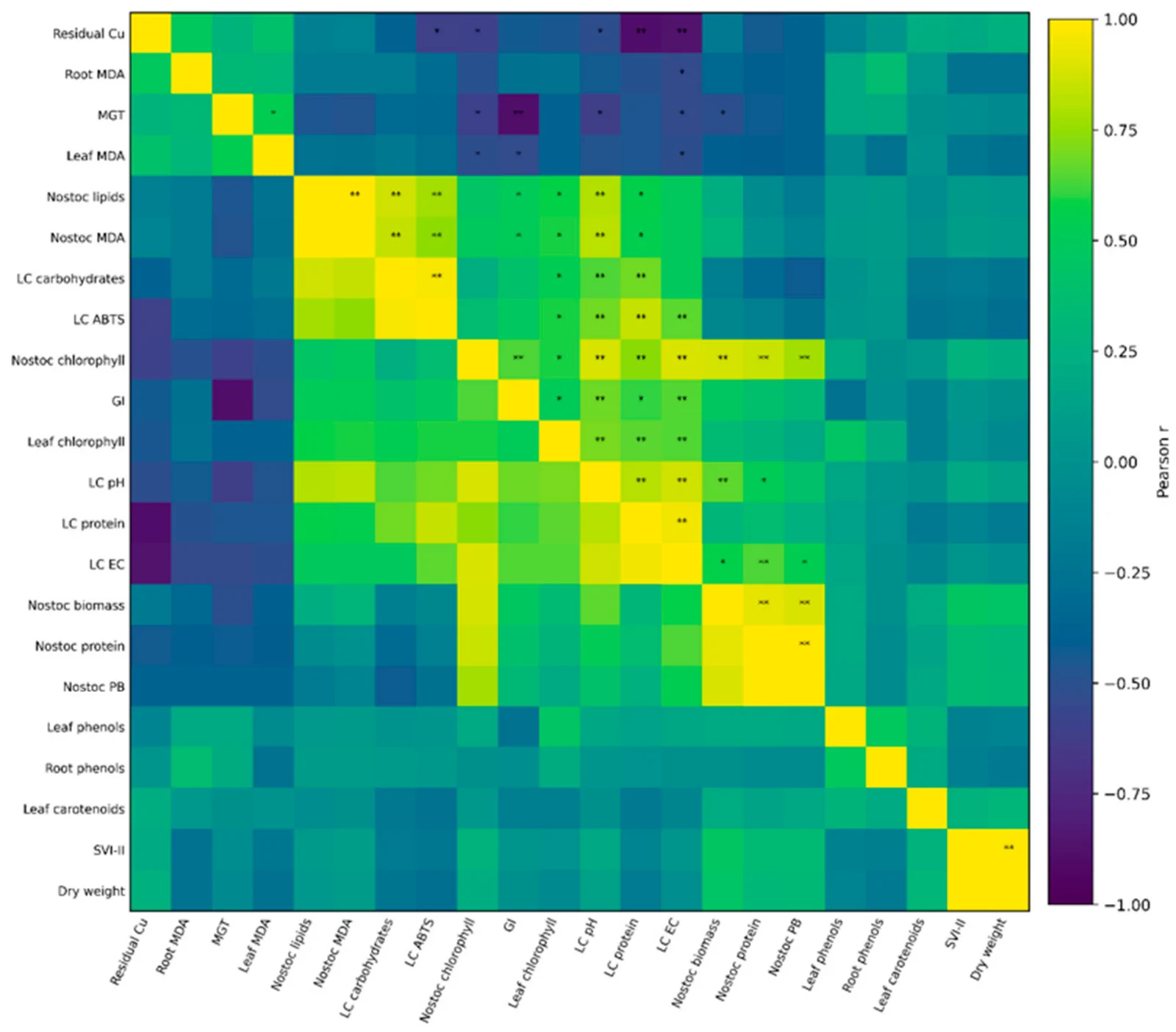
Clustered Pearson correlation matrix showing relationships among cyanobacterial physiological traits, culture liquid characteristics, and maize seedling responses. Asterisks indicate significant correlations (* *p* < 0.05, ** *p* < 0.01).

**Figure 4:**
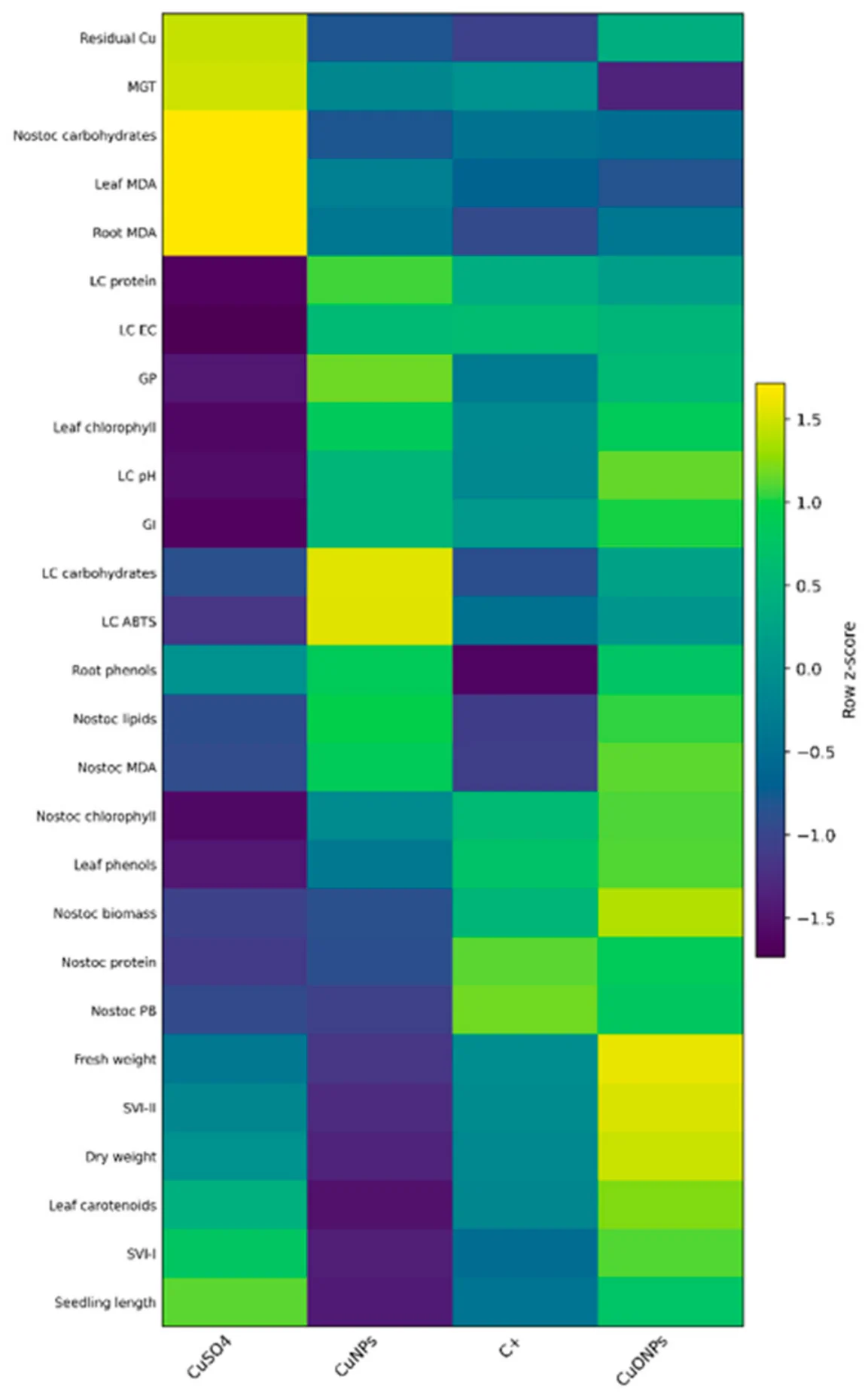
Integrated, clustered heat map illustrating coordinated changes in cyanobacterial physiology, culture fluid composition, and corn seedling responses under different culture fluid treatments after growing Nostoc treated with different forms of copper. Treatment: C−, deionized water; C+, culture liquid from control Nostoc; CuNPs, CuONPs, CuSO_4_—culture liquids from Nostoc grown with corresponding copper forms.

**Figure 5:**
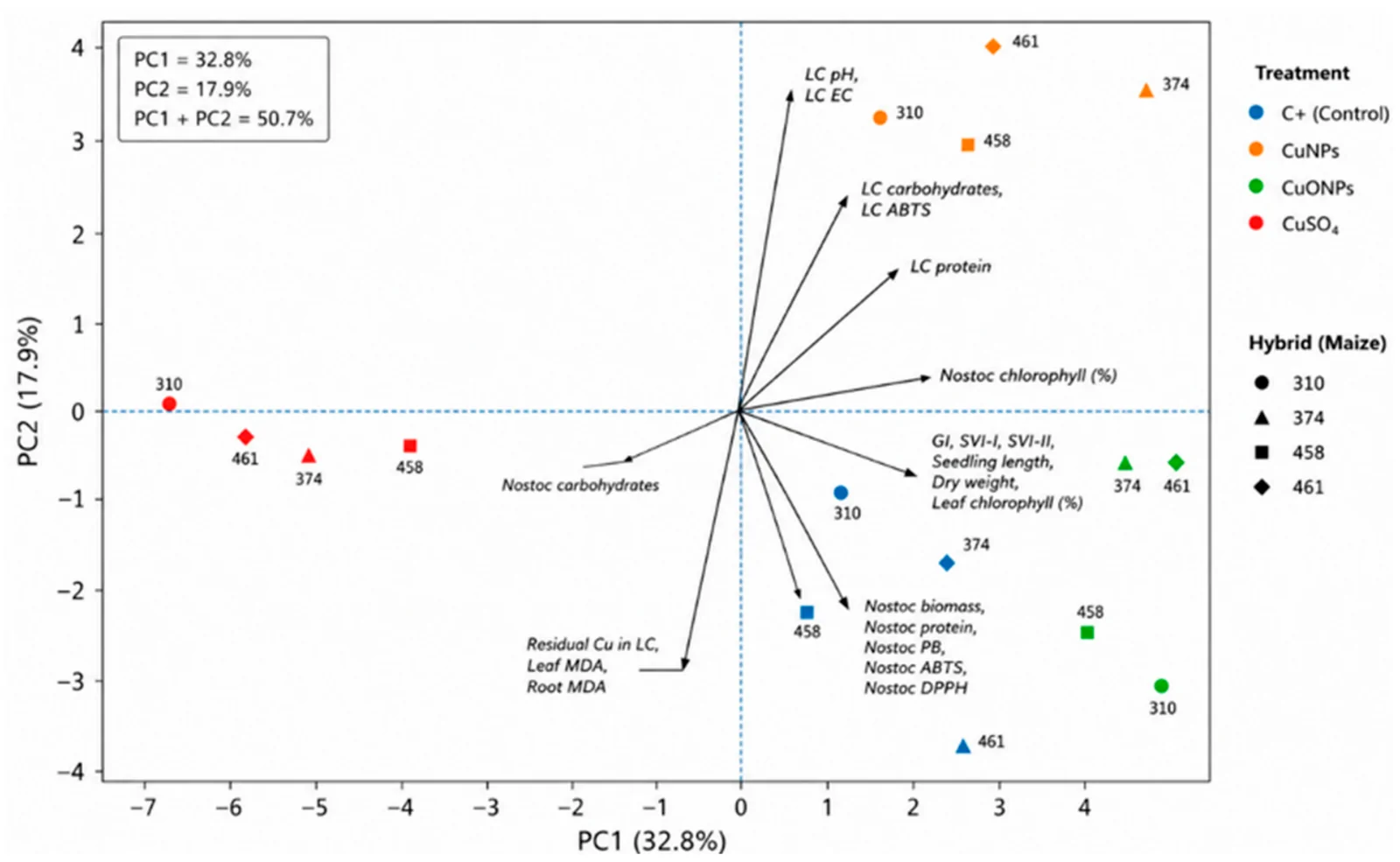
Integrated PCA biplot (simplified). Variables are shown as loading vectors. Only key variable groups are labeled for clarity.

Although the preceding results demonstrated that the chemical form of copper influences the physiology of *Nostoc linckia*, the biochemical composition of the culture liquid, and the response of maize seedlings, analysis of these datasets individually does not reveal the functional relationships among these processes. Therefore, an integrated analysis using a correlation matrix, a heat map, and principal component analysis (PCA) was performed to elucidate the functional links among the cyanobacterial response, the biochemical remodeling of the culture liquid, and the physiological effects observed in maize seedlings (Figure 3, Figure 4 and Figure 5).

Correlation analysis revealed a strong association among the physiological status of *Nostoc linckia*, the biochemical composition of the culture liquid, and the subsequent response of maize seedlings (Figure 3). Cyanobacterial biomass accumulation, protein content, chlorophyll, and phycobiliprotein levels were all positively correlated with germination index, seedling vigor, seedling dry biomass, and chlorophyll accumulation in the leaves. Likewise, extracellular protein content, carbohydrate concentration, and the antioxidant activity of the culture liquid showed positive correlations with seedling performance parameters. These relationships suggest that the metabolic changes in *Nostoc linckia* were associated with changes in the biochemical composition of the culture liquid, which in turn were associated with the physiological responses observed in maize seedlings. These observations are consistent with the hypothesis that the extracellular fraction contributes to the observed biostimulatory effects.

In contrast, residual copper concentration showed weak or negative correlations with most parameters related to plant growth and photosynthetic performance but was positively associated with markers of lipid peroxidation. Thus, the residual copper concentration was not significantly associated with the principal germination parameters, including the germination index (GI; r = −0.433, *p* = 0.094) and mean germination time (MGT; r = 0.278, *p* = 0.298), nor with leaf chlorophyll content (r = −0.452, *p* = 0.079). A significant positive correlation was observed only with seedling length (r = 0.534, *p* = 0.033), whereas the correlation with root MDA was close to, but did not reach, statistical significance (r = 0.488, *p* = 0.055). These findings indicate that residual copper alone cannot account for the overall pattern of germination and seedling responses observed in this study. Nevertheless, its contribution to specific plant traits cannot be excluded. Overall, the correlation matrix shows that changes in cyanobacterial metabolism are consistently reflected in both the culture liquid’s biochemical composition and the physiological responses of maize seedlings.

Integrated hierarchical clustering further revealed distinct metabolic profiles associated with each copper treatment (Figure 4). Among all treatments, the culture liquid obtained from *Nostoc linckia* cultivated in the presence of CuONPs exhibited the most balanced metabolic profile. This treatment combined high cyanobacterial biomass productivity with the maintenance of protein and photosynthetic pigment contents in the biomass, enhanced extracellular carbohydrate production, and improved the performance of primed maize seedlings, including higher vigor indices, greater biomass accumulation, and more favorable biochemical characteristics. In contrast, the CuSO_4_ treatment elicited a distinct response profile, characterized by reduced cyanobacterial productivity, lower extracellular proteins levels, and less favorable biochemical responses in maize seedlings. CuNP treatment occupied an intermediate position, sharing several beneficial characteristics with CuONP treatment while also exhibiting stronger oxidative stress in cyanobacterial cells.

Principal component analysis (PCA) integrated the responses obtained across the entire experimental system and revealed coordinated response patterns (Figure 5). The first two principal components together explained 50.7% of the total variance, clearly separating the experimental treatments according to their integrated biological effects. The remaining variance was distributed among several subsequent principal components (PC3–PC6), each explaining less than 11.5% of the total variance. This indicates that this variation was not associated with a single dominant biological gradient but rather reflected the complexity of the integrated physiological and biochemical dataset.

Samples associated with CuONPs formed a distinct cluster from those treated with CuSO_4_, whereas the control and CuNP-derived culture liquid occupied intermediate positions. The distribution of the loading vectors indicated that cyanobacterial biomass, protein content, photosynthetic pigments, extracellular proteins, and carbohydrates, together with maize seedling vigor indices and chlorophyll content, contributed positively to the same principal component, whereas residual copper concentration and lipid peroxidation markers were negatively associated with it. This configuration suggests that improvements in maize physiology were associated with coordinated metabolic changes occurring during cyanobacterial cultivation. These findings support the existence of a functional continuum linking cyanobacterial metabolism, the biochemical composition of the culture medium, and the physiological response of plants.

Although correlation and multivariate analyses cannot establish causality, the convergence of independent datasets supports the proposed biological interpretation and identifies coordinated response patterns across the experimental system. Taken together, these multivariate analyses provide experimental evidence supporting the central hypothesis of the present study. The different chemical forms of copper elicited distinct metabolic responses in *Nostoc linckia*, resulting in both quantitative and qualitative changes in the culture liquid. These alterations were subsequently reflected in seed germination, seedling development, and the biochemical responses of maize seedlings. The integrated analyses further indicate that CuONPs represent the most promising candidate for the controlled metabolic modulation of *Nostoc linckia*. Unlike ionic copper, which predominantly inhibited cyanobacterial metabolism, CuONPs induced moderate oxidative stress without compromising growth or photosynthetic activity, thereby enhancing the biostimulatory properties of culture liquid.

Considering all of the findings, it can be concluded that the efficacy of biostimulant is more closely associated with the metabolic remodeling of *Nostoc linckia* induced by different chemical forms of copper rather than on the residual copper concentration itself. Therefore, the controlled modulation of cyanobacterial metabolism using CuONPs may provide a basis for a metabolic elicitation strategy to develop a new generation of microbial biostimulants with enhanced functional properties.

## 5. Study Limitations

This study was conducted under controlled laboratory conditions using a single cyanobacterial strain (*Nostoc linckia* CNMN-CB-03) and one concentration of three copper forms. Although four maize hybrids were included to account for genotype-dependent responses, the observed early biostimulant effects should be further validated under greenhouse and field conditions. In addition, the economic feasibility, production scalability, storage stability, and practical applicability of the culture liquid remain to be established. Another limitation is that the molecular and biochemical mechanisms underlying the observed adaptive responses were not directly investigated. Consequently, the proposed involvement of hormesis and stress adaptation is inferred from physiological and biochemical evidence rather than from direct characterization of antioxidant defense pathways, signaling networks, or gene expression. Likewise, the molecular basis of the genotype-dependent responses among maize hybrids remains to be elucidated.

The extracellular composition of the culture liquid was characterized using biochemical assays and FTIR spectroscopy rather than targeted metabolomic analyses; therefore, the specific bioactive compounds responsible for the observed plant responses remain to be identified. Future studies integrating metabolomic and transcriptomic approaches would provide deeper insight into copper-induced metabolic reprogramming and the molecular mechanisms responsible for the biostimulant activity of cyanobacterial culture liquids. Finally, residual copper concentrations were determined only at the end of cultivation. Time-resolved monitoring throughout the cultivation period would provide a more comprehensive understanding of copper transformation, uptake dynamics, and their contribution to the observed biological responses.

## 6. Conclusions

The present study demonstrates that the physiological and metabolic responses of *Nostoc linckia* depend primarily on its chemical form. Although identical copper concentrations were applied, CuNPs, CuONPs, and CuSO_4_ induced markedly different physiological and metabolic responses.

Among the tested treatments, CuONPs provided the most balanced overall metabolic response, combining high biomass productivity with the maintenance of protein and photosynthetic pigment contents while activating adaptive oxidative responses. Cultures exposed to CuONPs exhibited high biomass productivity, elevated levels of photosynthetic pigments and proteins, and increased antioxidant activity while simultaneously activating antioxidant defense mechanisms.

Copper-induced metabolic remodeling was reflected not only in the biochemical composition of the cyanobacterial biomass, but also in the characteristics of the culture liquid. The altered profile of culture liquid was reflected in genotype-dependent differences in maize seed germination dynamics, seedling vigor, and biochemical composition.

Integrated analyses of the complete experimental dataset revealed that improvements in maize performance were closely associated with coordinated changes in cyanobacterial physiology and extracellular metabolism.

Overall, the results demonstrate that the chemical form of copper is a key determinant of metabolic remodeling in *Nostoc linckia* and of the biological properties of both the cyanobacterial biomass and the culture liquid. Among the investigated treatments, CuONPs exhibited the most favorable metabolic profile and the greatest biostimulatory activity toward maize seedlings. These findings provide a foundation for developing controlled metabolic elicitation strategies to optimize cyanobacterial culture liquid as promising sources of next-generation microbial biostimulants with enhanced functional properties.

## Figures and Tables

**Table 1:** Dissolution of CuNPs and CuONPs in sterile mineral medium under the physicochemical conditions used in the biological experiments.

Time	CuNPs (mg/L)	CuONPs (mg/L)
6 h	0.0095 ± 0.0008	0.0785 ± 0.0024
12 h	0.0144 ± 0.0005	0.0788 ± 0.0069
24 h	0.0809 ± 0.0054	0.0683 ± 0.0027
36 h	0.0822 ± 0.0052	0.0469 ± 0.0029
48 h	0.0816 ± 0.0083	0.0434 ± 0.0034
72 h	0.0872 ± 0.0072	0.0508 ± 0.0023

**Table 2:** Biomass, biochemical composition, and antioxidant capacity of *Nostoc linckia* cultivated in the presence of different copper forms *.

Parameter	Control	CuNPs	CuONPs	CuSO_4_
Biomass (g/L)	0.804 ± 0.011 ^b^	0.608 ± 0.011 ^c^	0.925 ± 0.008 ^a^	0.588 ± 0.021 ^c^
Proteins (%)	22.880 ± 0.532 ^a^	13.840 ± 0.159 ^c^	21.750 ± 0.201 ^b^	12.860 ± 0.337 ^d^
Carbohydrates (%)	28.260 ± 1.060 ^b^	27.604 ± 0.786 ^b^	28.150 ± 1.585 ^b^	32.220 ± 1.060 ^a^
Chlorophyll a (%)	0.425 ± 0.003 ^b^	0.379 ± 0.005 ^c^	0.460 ± 0.004 ^a^	0.280 ± 0.004 ^d^
Phycobiliproteins (%)	13.450 ± 0.134 ^a^	2.920 ± 0.019 ^d^	11.550 ± 0.060 ^b^	3.440 ± 0.076 ^c^
Lipids (%)	2.072 ± 0.189 ^b^	3.660 ± 0.208 ^a^	3.710 ± 0.067 ^a^	2.222 ± 0.038 ^b^
MDA (nM/g)	3.120 ± 0.076 ^d^	6.880 ± 0.121 ^b^	7.410 ± 0.105 ^a^	3.440 ± 0.060 ^c^
DPPH (% inhibition)	36.450 ± 1.908 ^b^	29.580 ± 1.787 ^c^	40.370 ± 2.534 ^a^	30.150 ± 0.859 ^c^
ABTS (% inhibition)	42.546 ± 3.033 ^a^	22.490 ± 1.082 ^c^	28.320 ± 2.077 ^b^	20.540 ± 1.563 ^c^

* Note: Different letters in the exponent within the same row indicate significant differences between treatments, according to the post-hoc Tukey HSD test (*p* < 0.05).

**Table 3:** pH, electrical conductivity, and residual copper concentration in the culture liquid of *Nostoc linckia* cultivated in the presence of different copper forms *.

Experimental Variant	pH	Electrical Conductivity (µS cm^−1^)	Residual Cu (mg L^−1^)
Control	9.15 ± 0.12 ^c^	1640 ± 21 ^a^	BDL ^a^
CuNPs	9.38 ± 0.09 ^b^	1621 ± 13 ^a^	0.16 ± 0.04 ^a^
CuONPs	9.63 ± 0.11 ^a^	1620 ± 9 ^a^	0.96 ± 0.10 ^b^
CuSO_4_	8.65 ± 0.08 ^d^	1528 ± 16 ^b^	1.64 ± 0.27 ^c^

* Note: Values are presented as mean ± SD, n = 5. Different superscript letters within the same column indicate significant differences (one-way ANOVA + Tukey HSD, *p* < 0.05).

**Table 4:** Effect of culture liquid priming on the seed germination of four maize hybrids *.

Hybrid	Treatment	GP (%)	MGT (h)	GI
374	C−	86.00 ± 4.18 ^a^	21.35 ± 1.04 ^ac^	0.88 ± 0.08 ^bc^
	C+	90.00 ± 7.07 ^a^	20.80 ± 1.20 ^bc^	0.99 ± 0.12 ^bd^
	CuNPs	92.00 ± 5.70 ^a^	18.96 ± 1.82 ^bd^	1.14 ± 0.04 ^a^
	CuONPs	89.00 ± 6.52 ^a^	18.18 ± 0.54 ^d^	1.12 ± 0.07 ^ad^
	CuSO_4_	85.00 ± 5.00 ^a^	23.42 ± 0.91 ^a^	0.75 ± 0.05 ^c^
310	C−	93.00 ± 6.71 ^a^	22.38 ± 1.73 ^a^	0.91 ± 0.06 ^a^
	C+	86.00 ± 4.18 ^a^	22.42 ± 1.48 ^a^	0.83 ± 0.04 ^a^
	CuNPs	95.00 ± 3.54 ^a^	23.10 ± 1.44 ^a^	0.91 ± 0.08 ^a^
	CuONPs	92.00 ± 5.70 ^a^	21.54 ± 2.83 ^a^	0.89 ± 0.11 ^a^
	CuSO_4_	93.00 ± 8.37 ^a^	24.33 ± 0.99 ^a^	0.80 ± 0.06 ^a^
458	C−	100.00 ± 0.00 ^a^	22.44 ± 0.68 ^ab^	0.96 ± 0.06 ^ab^
	C+	98.00 ± 2.74 ^a^	21.56 ± 0.72 ^b^	0.99 ± 0.07 ^a^
	CuNPs	97.00 ± 2.74 ^a^	22.50 ± 1.07 ^ab^	0.92 ± 0.05 ^ab^
	CuONPs	98.00 ± 2.74 ^a^	22.65 ± 0.52 ^ab^	0.92 ± 0.02 ^ab^
	CuSO_4_	97.00 ± 4.47 ^a^	23.61 ± 0.73 ^a^	0.86 ± 0.06 ^b^
461	C−	93.00 ± 4.47 ^a^	22.41 ± 1.20 ^ab^	0.94 ± 0.05 ^b^
	C+	98.00 ± 4.47 ^a^	23.89 ± 1.31 ^a^	0.93 ± 0.10 ^b^
	CuNPs	96.00 ± 4.18 ^a^	23.34 ± 1.20 ^a^	0.90 ± 0.04 ^b^
	CuONPs	98.00 ± 2.74 ^a^	20.31 ± 0.93 ^b^	1.10 ± 0.02 ^a^
	CuSO_4_	91.00 ± 8.22 ^a^	23.91 ± 1.21 ^a^	0.79 ± 0.04 ^c^

* Note: Values are presented as mean ± SD (n = 5). Treatment: C−, deionized water; C+, culture liquid from control Nostoc; CuNPs, CuONPs, CuSO_4_—culture liquids from Nostoc grown with corresponding copper forms. Different superscript letters within the same hybrid and parameter indicate significant differences among priming treatments according to Tukey’s HSD post hoc test (*p* < 0.05).

**Table 5:** Effect of seed priming on seedling growth and vigor of maize hybrids *.

Hybrid	Treatment	Seedling Length (cm)	Fresh Weight (g)	Dry Weight (g)	SVI-I	SVI-II
374	C−	17.63 ± 1.46 ^ab^	0.33 ± 0.06 ^a^	0.06 ±0.01 ^ab^	1520.02 ± 185.77 ^a^	4.91 ± 0.74 ^a^
	C+	15.00 ± 2.17 ^c^	0.25 ± 0.05 ^a^	0.05 ± 0.01 ^ab^	1362.19 ± 289.93 ^a^	4.55 ± 0.82 ^a^
	CuNPs	15.90 ± 0.74 ^bc^	0.27 ± 0.05 ^a^	0.05 ± 0.00 ^b^	1460.30 ± 50.75 ^a^	4.59 ± 0.42 ^a^
	CuONPs	17.32 ± 1.32 ^abc^	0.27 ± 0.06 ^a^	0.06 ± 0.01 ^ab^	1543.37 ± 185.89 ^a^	4.93 ± 0.94 ^a^
	CuSO_4_	19.23 ± 0.71 ^a^	0.25 ± 0.03 ^a^	0.06 ± 0.00 ^a^	1634.40 ± 106.78 ^a^	5.25 ± 0.59 ^a^
310	C−	18.08 ± 2.53 ^a^	0.23 ± 0.04 ^b^	0.04 ± 0.01 ^b^	1690.70 ± 322.15 ^a^	3.84 ± 0.54 ^b^
	C+	16.80 ± 3.07 ^a^	0.20 ± 0.03 ^b^	0.04 ± 0.01 ^b^	1449.50 ± 301.94 ^a^	3.17 ± 0.36 ^b^
	CuNPs	17.34 ± 2.66 ^a^	0.23 ± 0.07 ^b^	0.04 ± 0.01 ^b^	1652.80 ± 294.64 ^a^	4.02 ± 1.10 ^b^
	CuONPs	18.28 ± 3.30 ^a^	0.42 ± 0.07 ^a^	0.08 ± 0.01 ^a^	1683.20 ± 322.99 ^a^	7.10 ± 1.34 ^a^
	CuSO_4_	20.00 ± 1.15 ^a^	0.23 ± 0.05 ^b^	0.04 ± 0.01 ^b^	1867.40 ± 266.34 ^a^	3.83 ± 1.00 ^b^
458	C−	16.34 ± 1.23 ^b^	0.23 ± 0.04 ^b^	0.04 ± 0.01 ^b^	1634.20 ± 123.28 ^bc^	4.34 ± 0.77 ^b^
	C+	19.88 ± 1.57 ^a^	0.24 ± 0.03 ^bc^	0.04 ± 0.01 ^b^	1946.55 ± 126.08 ^a^	4.28 ± 0.56 ^b^
	CuNPs	16.30 ± 1.58 ^b^	0.24 ± 0.03 ^bc^	0.04 ± 0.01 ^b^	1582.16 ± 177.76 ^c^	4.19 ± 0.64 ^b^
	CuONPs	19.49 ± 1.47 ^a^	0.35 ± 0.05 ^a^	0.06 ± 0.01 ^a^	1908.51 ± 120.55 ^ab^	6.29 ± 0.84 ^a^
	CuSO_4_	17.79 ± 1.97 ^ab^	0.30 ± 0.03 ^ac^	0.05 ± 0.01 ^ab^	1723.12 ± 178.19 ^abc^	5.24 ± 0.57 ^ab^
461	C−	19.58 ± 2.69 ^a^	0.22 ± 0.03 ^b^	0.04 ± 0.01 ^b^	1819.40 ± 257.32 ^a^	3.80 ± 0.52 ^b^
	C+	19.27 ± 1.63 ^a^	0.38 ± 0.07 ^a^	0.07 ± 0.01 ^a^	1884.60 ± 131.50 ^a^	6.82 ± 1.35 ^a^
	CuNPs	17.48 ± 1.89 ^a^	0.18 ± 0.02 ^b^	0.03 ± 0.00 ^b^	1674.15 ± 143.14 ^a^	3.18 ± 0.24 ^b^
	CuONPs	20.40 ± 1.19 ^a^	0.25 ± 0.04 ^b^	0.05 ± 0.01 ^b^	2000.01 ± 151.32 ^a^	4.46 ± 0.65 ^b^
	CuSO_4_	19.92 ± 1.71 ^a^	0.26 ± 0.07 ^b^	0.05 ± 0.01 ^b^	1812.10 ± 216.39 ^a^	4.34 ± 1.27 ^b^

* Note: Values are presented as mean ± SD (n = 5). Treatment: C−, deionized water; C+, culture liquid from control Nostoc; CuNPs, CuONPs, CuSO_4_—culture liquids from Nostoc grown with corresponding copper forms. Different superscript letters within the same hybrid and parameter indicate significant differences among priming treatments according to Tukey’s HSD post hoc test (*p* < 0.05).

## Data Availability

The original contributions presented in this study are included in the article. Further inquiries are directed to the corresponding author.

## Supplementary Materials

The following supporting information can be downloaded at: https://www.mdpi.com/article/10.3390/life16081302/s1, Figure S1: Germination and early seedling development experiment (representative images); Figure S2: FTIR spectra of culture liquid obtained from *Nostoc linckia* cultivated in the presence of different copper forms; Figure S3: Normalized FTIR spectra in the fingerprint region (1800–800 cm^−1^) of the lyophilized culture liquids; Table S1: Results of one-way ANOVA; Table S2: Results of two-way ANOVA; Table S3: Biochemical characteristics of the leaves and roots of maize seedlings after seed priming with culture liquid.
