# Supplementary material for: Comparative Effects of CuNPs, CuONPs and CuSO4 on Biomass Quality, Culture Liquid Composition and Biostimulant Activity of Nostoc linckia
Source: Life (Basel). 2026 Aug 8;16(8):1302. doi: 10.3390/life16081302 (PMC13514169; doi:10.3390/life16081302)
Supplement: Supplementary file 1 [file life-16-01302-s001.zip › Figure S1. Germination and early seedling development experience (representative images).pdf]

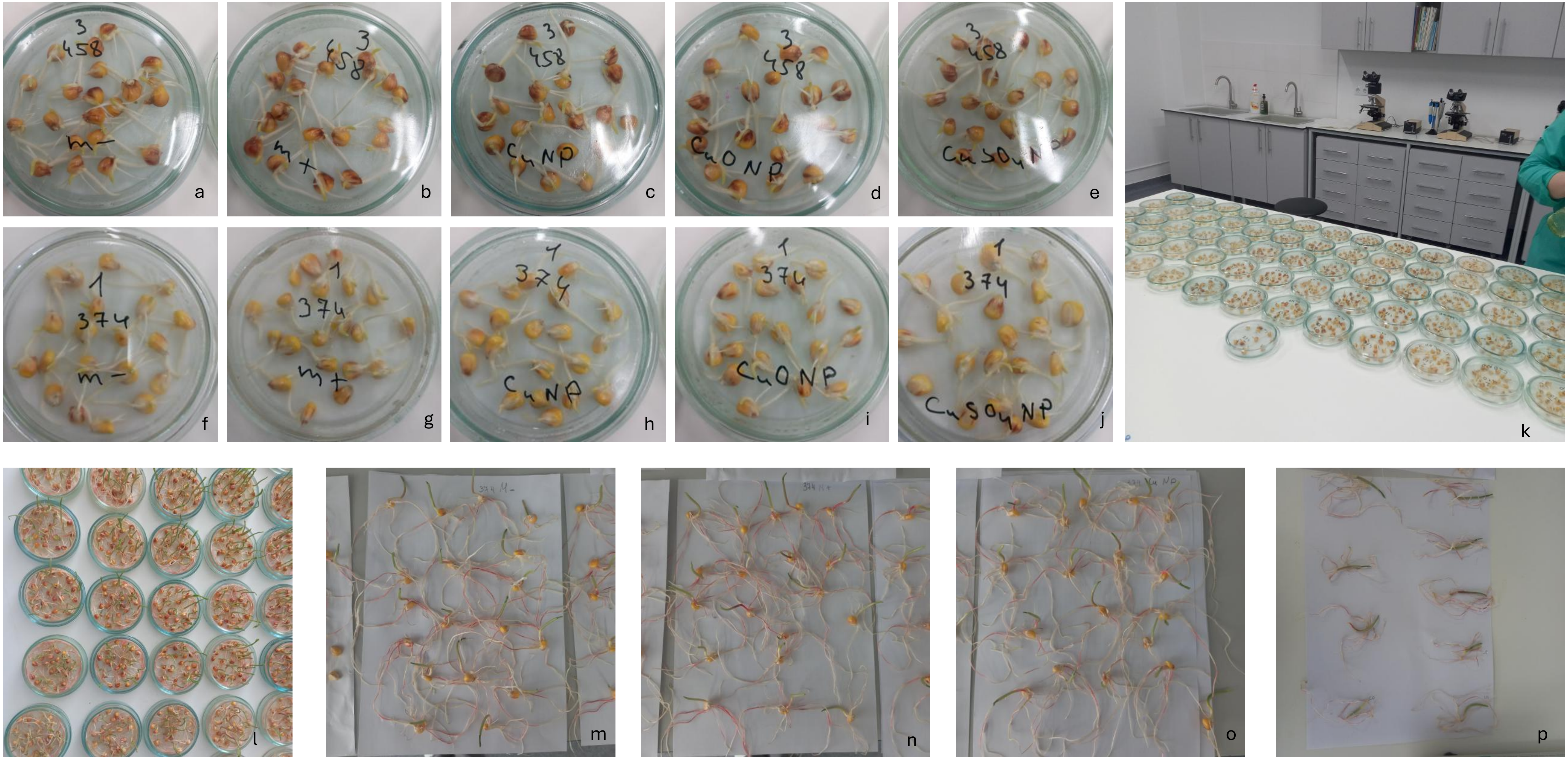

Figure S1. Germination and early seedling development experience (representative images). a-e - hybrid 458, germination, 24 hours; f-j hybrid 374, germination 24 hours; k - samples prepared for germination counting; l - appearance of seedlings before preparation for analyses; m-o –seedlings prepared for fresh biomass determination; p - seedlings separated into shoots and roots prior to biometric and biochemical analyses
