# Supplementary material for: Comparative Effects of CuNPs, CuONPs and CuSO4 on Biomass Quality, Culture Liquid Composition and Biostimulant Activity of Nostoc linckia
Source: Life (Basel). 2026 Aug 8;16(8):1302. doi: 10.3390/life16081302 (PMC13514169; doi:10.3390/life16081302)
Supplement: Supplementary file 1 [file life-16-01302-s001.zip › Figure S2. FTIR spectra of culture liquid obtained from Nostoc linckia cultivated in the presence of different copper forms.pdf]

Figure S2. FTIR spectra of culture liquid obtained from *Nostoc linckia* cultivated in the presence of different copper forms

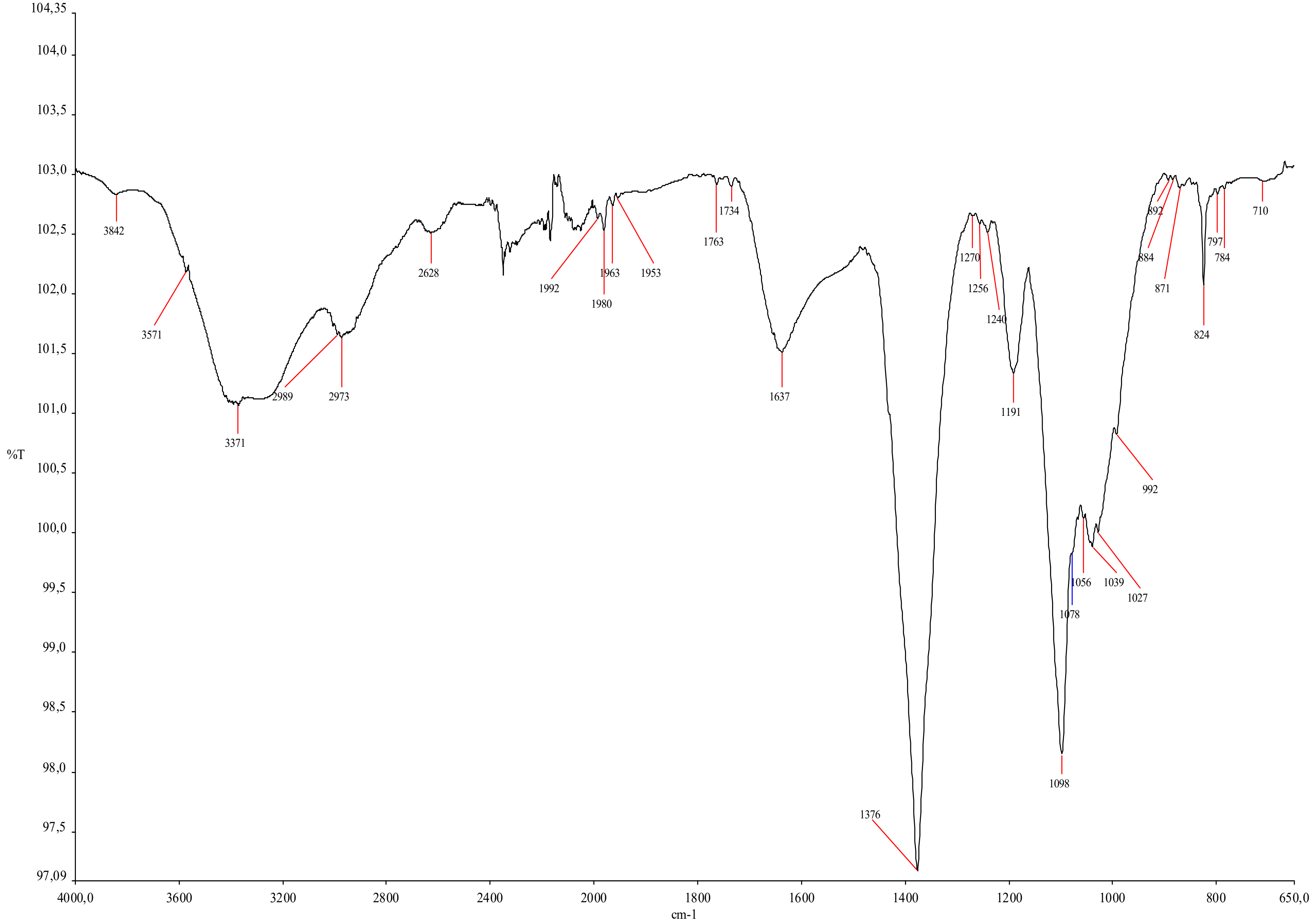

A, FTIR spectrum of the culture liquid from of the control

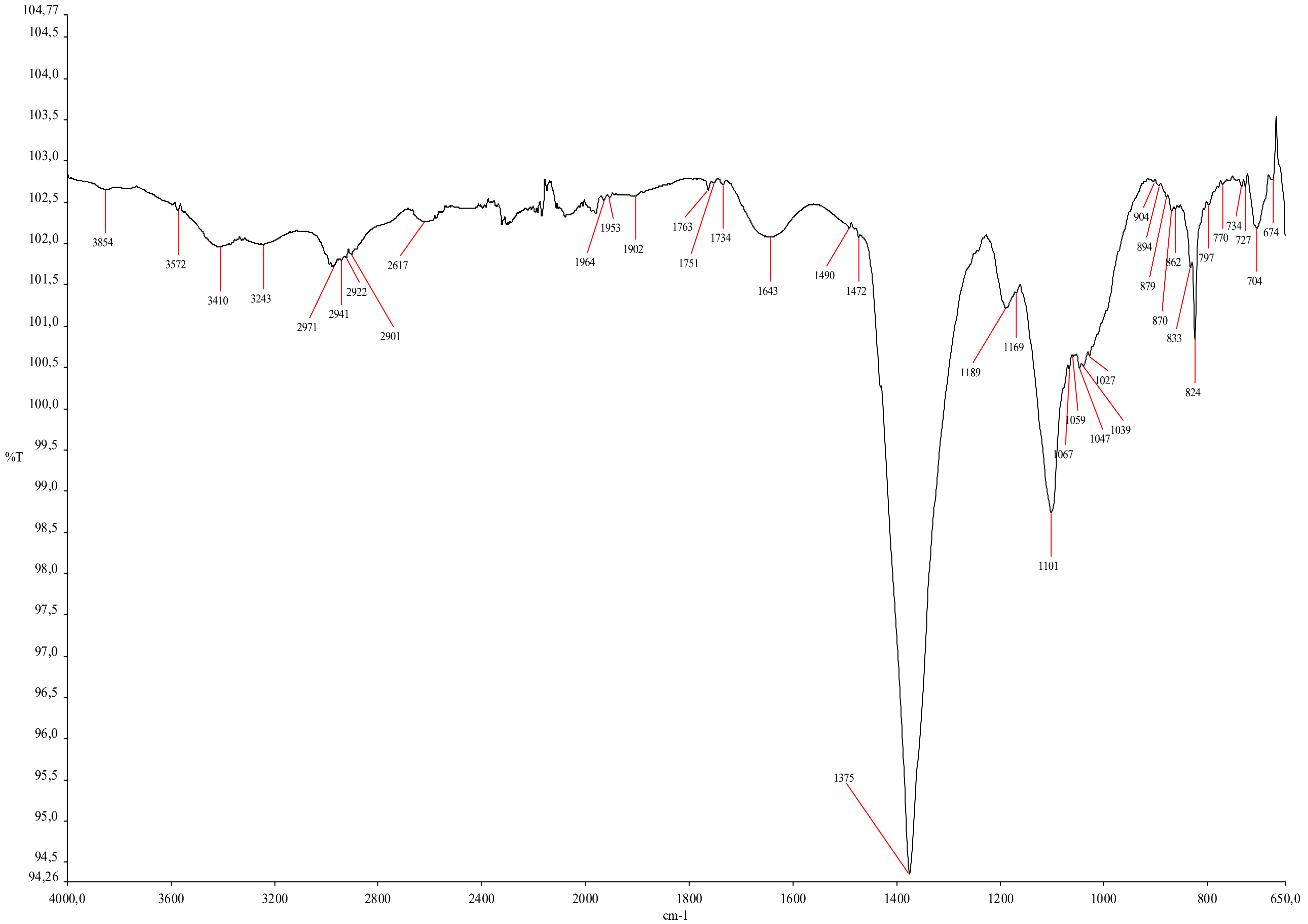

B, FTIR spectrum of the culture liquid from the growth of Nostoc with the addition of CuNPs

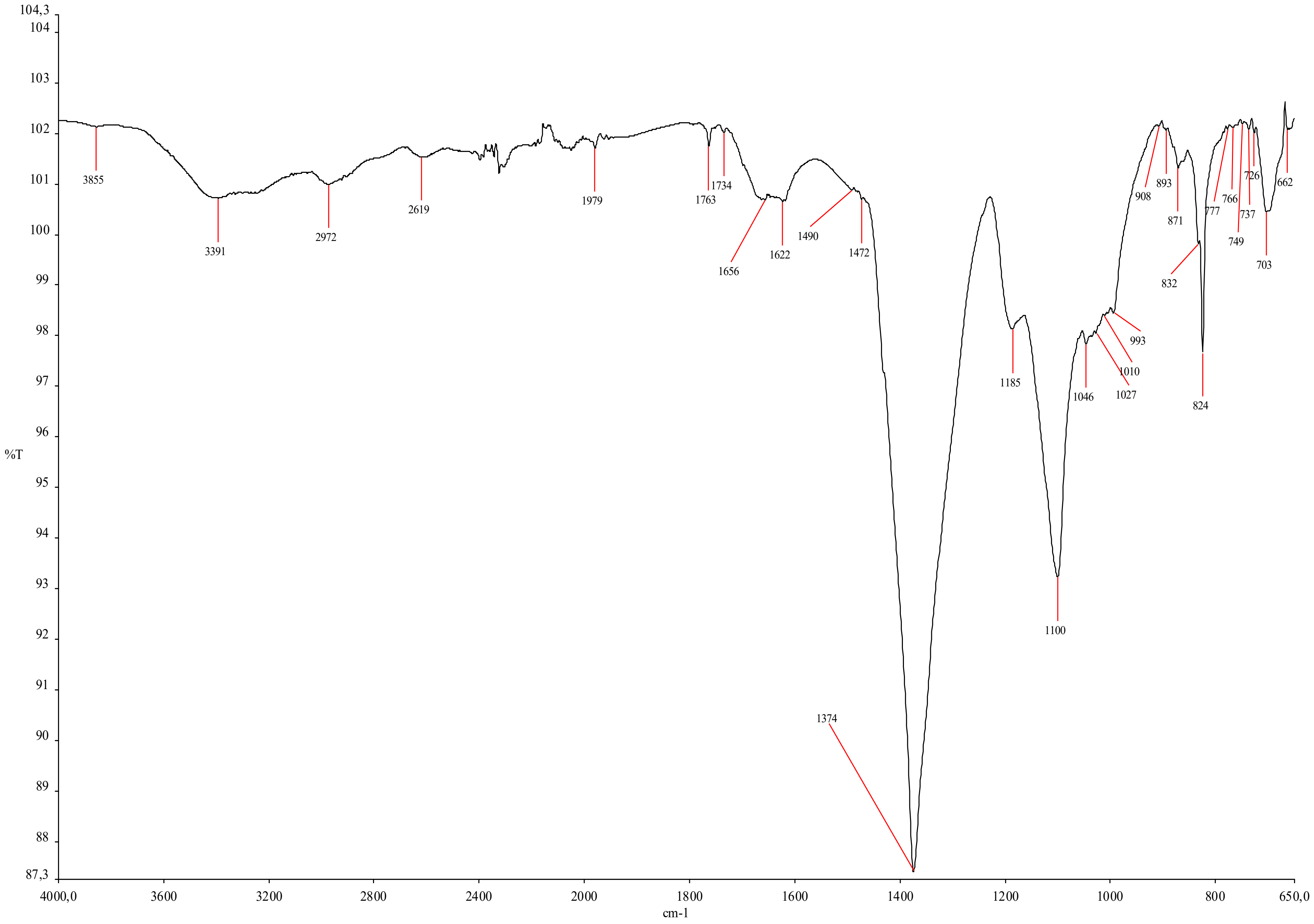

C, FTIR spectrum of the culture liquid from the growth of Nostoc with the addition of CuONPs

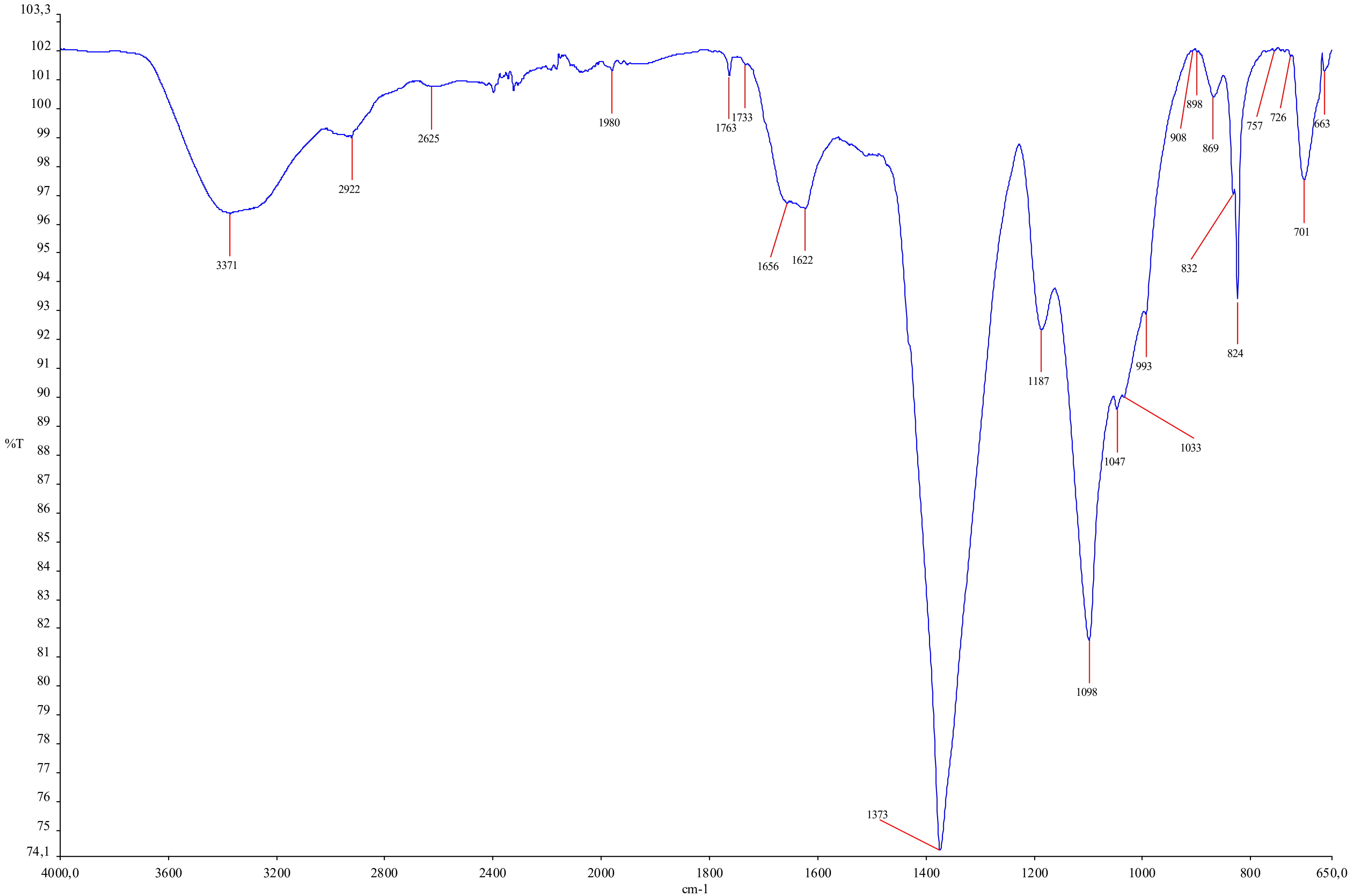

D, FTIR spectrum of the culture liquid from the growth of Nostoc with the addition of $CuSO_4 \cdot 5H_2O$
