# Supplementary material for: Comparative Effects of CuNPs, CuONPs and CuSO4 on Biomass Quality, Culture Liquid Composition and Biostimulant Activity of Nostoc linckia
Source: Life (Basel). 2026 Aug 8;16(8):1302. doi: 10.3390/life16081302 (PMC13514169; doi:10.3390/life16081302)
Supplement: Supplementary file 1 [file life-16-01302-s001.zip › Table S1. Results of one-way ANOVA.pdf]

Table S1. Results of one-way ANOVA for physiological, biochemical and culture liquid characteristics of *Nostoc linckia* cultivated in the presence of different copper forms.

Parameter	df (between, within)	F	p-value
**Biomass (g L^{-1})**	3, 16	724.23	<0.001
**Proteins (%)**	3, 16	1173.23	<0.001
**Carbohydrates (%)**	3, 16	16.82	<0.001
**Chlorophyll *a***	3, 16	1896.51	<0.001
**Phycobiliproteins (%)**	3, 16	21295.59	<0.001
**Lipids (%)**	3, 16	186.64	<0.001
**MDA (nM g^{-1})**	3, 16	2873.67	<0.001
**DPPH (% inhibition)**	3, 16	38.54	<0.001
**ABTS (% inhibition)**	3, 16	115.50	<0.001
**Extracellular proteins**	3, 16	250.44	<0.001
**Extracellular carbohydrates**	3, 16	12.17	<0.001
**Extracellular ABTS**	3, 16	16.84	<0.001
**pH**	3, 16	116.69	<0.001
**Electrical conductivity**	3, 16	13.23	<0.001
**Residual Cu**	3, 16	201.27	<0.001

*All analyses compared 4 treatments (C, CuNPs, CuONPs, $CuSO_4$), (n = 5 biological replicates per treatment)
