# Supplementary material for: Comparative Effects of CuNPs, CuONPs and CuSO4 on Biomass Quality, Culture Liquid Composition and Biostimulant Activity of Nostoc linckia
Source: Life (Basel). 2026 Aug 8;16(8):1302. doi: 10.3390/life16081302 (PMC13514169; doi:10.3390/life16081302)
Supplement: Supplementary file 1 [file life-16-01302-s001.zip › Table S2. Results of two-way ANOVA.pdf]

Table S2. Results of two-way ANOVA for maize germination, seedling growth and biochemical parameters following seed priming with culture liquids obtained from *Nostoc linckia* cultivated in the presence of different copper forms

Parameter	Source	df	F	p-value
**Fresh weight (g)**	Hybrid	3	0.58	0.628
	Treatment	4	10.78	<0.001
	Hybrid × Treatment	12	9.12	<0.001
**Dry weight (g)**	Hybrid	3	4.50	0.006
	Treatment	4	14.94	<0.001
	Hybrid × Treatment	12	9.68	<0.001
**Seedling length (cm)**	Hybrid	3	6.00	<0.001
	Treatment	4	5.15	<0.001
	Hybrid × Treatment	12	1.95	0.040
**GP (%)**	Hybrid	3	16.65	<0.001
	Treatment	4	1.38	0.247
	Hybrid × Treatment	12	1.54	0.128
**MGT (h)**	Hybrid	3	17.62	<0.001
	Treatment	4	15.10	<0.001
	Hybrid × Treatment	12	3.18	<0.001
**GI**	Hybrid	3	12.01	<0.001
	Treatment	4	27.69	<0.001
	Hybrid × Treatment	12	7.44	<0.001
**SVI-I**	Hybrid	3	11.33	<0.001
	Treatment	4	2.70	0.036
	Hybrid × Treatment	12	1.78	0.067
**SVI-II**	Hybrid	3	2.08	0.110
	Treatment	4	12.61	<0.001
	Hybrid × Treatment	12	8.58	<0.001
**Leaf ABTS (% inhibition)**	Hybrid	3	176.67	<0.001
	Treatment	4	1.96	0.109
	Hybrid × Treatment	12	3.52	<0.001
**Leaf DPPH (% inhibition)**	Hybrid	3	247.45	<0.001
	Treatment	4	3.23	0.016
	Hybrid × Treatment	12	2.43	0.010
**Leaf phenols (mg GAE g^{-1})**	Hybrid	3	129.63	<0.001
	Treatment	4	3.89	0.006
	Hybrid × Treatment	12	4.31	<0.001
**Leaf MDA (nM g^{-1})**	Hybrid	3	23.19	<0.001

	Treatment	4	8.61	<0.001
	Hybrid × Treatment	12	3.07	0.001
**Leaf chlorophyll (%)**	Hybrid	3	5.03	0.003
	Treatment	4	11.51	<0.001
	Hybrid × Treatment	12	2.58	0.006
**Leaf carotenoids (%)**	Hybrid	3	2.99	0.036
	Treatment	4	10.74	<0.001
	Hybrid × Treatment	12	16.27	<0.001
**Root ABTS (% inhibition)**	Hybrid	3	16.31	<0.001
	Treatment	4	0.95	0.440
	Hybrid × Treatment	12	2.55	0.007
**Root DPPH (% inhibition)**	Hybrid	3	0.55	0.653
	Treatment	4	1.02	0.401
	Hybrid × Treatment	12	2.81	0.003
**Root phenols (mg GAE g^{-1})**	Hybrid	3	111.55	<0.001
	Treatment	4	6.02	<0.001
	Hybrid × Treatment	12	8.22	<0.001
**Root MDA (nM g^{-1})**	Hybrid	3	10.37	<0.001
	Treatment	4	6.08	<0.001
	Hybrid × Treatment	12	3.21	<0.001

*All analyses compared 5 treatments (C-, C+, CuNPs, CuONPs, CuSO4), (n = 5 biological replicates per treatment)
