# Supplementary material for: Comparative Effects of CuNPs, CuONPs and CuSO4 on Biomass Quality, Culture Liquid Composition and Biostimulant Activity of Nostoc linckia
Source: Life (Basel). 2026 Aug 8;16(8):1302. doi: 10.3390/life16081302 (PMC13514169; doi:10.3390/life16081302)
Supplement: Supplementary file 1 [file life-16-01302-s001.zip › Table S3. Biochemical characteristics of leaves and roots of maize seedlings after seed priming with culture liquid obtained fro.pdf]

Table S3. Biochemical characteristics of leaves and roots of maize seedlings after seed priming with culture liquid obtained from *Nostoc linckia* cultivated in the presence of different copper forms*.

Hybrid	***Treatment***	***Leaf ABTS (% inhibition)***	***Leaf DPPH (% inhibition)***	***Leaf phenols (mg GAE/g)***	***Leaf MDA (nM/g)***	***Leaf chlorophyll (%)***	***Leaf carotenoids (%)***	***Root ABTS (% inhibition)***	***Root DPPH (% inhibition)***	***Root phenols (mg GAE/g)***	***Root MDA (nM/g)***
**310**	C−	35.76 ± 3.69a	26.37 ± 2.42a	6.16 ± 0.48a	26.25 ± 3.18b	4.54 ± 0.63a	0.54 ± 0.18c	43.00 ± 6.97a	51.42 ± 6.84a	1.27 ± 0.34a	20.67 ± 4.83a
**310**	C+	38.15 ± 4.23a	28.69 ± 2.30a	7.00 ± 0.77a	22.16 ± 2.05b	4.98 ± 0.21a	0.38 ± 0.06c	42.36 ± 7.99a	51.72 ± 5.50a	1.27 ± 0.33a	16.15 ± 3.17b
**310**	CuNPs	28.45 ± 2.82b	22.17 ± 3.05b	5.02 ± 0.62b	19.28 ± 2.93c	5.29 ± 0.44a	0.10 ± 0.06c	42.17 ± 7.92a	43.82 ± 3.13a	1.35 ± 0.39a	23.13 ± 3.21a
**310**	CuONPs	33.78 ± 5.57a	24.52 ± 3.84a	6.50 ± 0.35a	19.69 ± 4.01c	5.24 ± 0.40a	1.76 ± 0.51a	43.37 ± 7.32a	47.91 ± 11.30a	1.40 ± 0.34a	15.13 ± 0.89b
**310**	CuSO4	29.71 ± 1.47b	21.79 ± 1.87b	5.12 ± 0.25b	32.61 ± 3.21a	3.70 ± 0.65b	1.07 ± 0.22b	42.91 ± 10.06a	50.08 ± 12.63a	1.25 ± 0.26a	27.18 ± 4.25a
**374**	C−	46.29 ± 5.84b	25.86 ± 4.17a	3.30 ± 0.64a	17.49 ± 2.70a	4.21 ± 0.44a	0.45 ± 0.26b	56.54 ± 10.03a	52.42 ± 8.94a	1.22 ± 0.29a	17.13 ± 4.00a
**374**	C+	49.49 ± 4.75a	28.00 ± 3.15a	3.50 ± 0.46a	17.18 ± 2.09a	4.19 ± 0.68a	1.24 ± 0.20a	52.77 ± 11.32a	47.61 ± 11.93a	1.03 ± 0.28a	18.05 ± 4.81a
**374**	CuNPs	54.40 ± 3.83a	30.99 ± 2.80a	3.77 ± 0.30a	16.05 ± 3.39a	4.99 ± 0.73a	0.41 ± 0.32b	51.40 ± 9.27a	44.99 ± 8.35a	1.04 ± 0.17a	16.26 ± 4.39a
**374**	CuONPs	54.17 ± 2.76a	30.11 ± 2.13a	3.63 ± 0.39a	16.26 ± 3.69a	4.72 ± 0.64a	0.62 ± 0.13b	54.14 ± 15.73a	53.86 ± 8.00a	1.18 ± 0.34a	15.90 ± 3.67a
**374**	CuSO4	54.80 ± 3.77a	29.83 ± 3.03a	3.76 ± 0.39a	20.67 ± 5.12a	3.11 ± 0.31b	0.77 ± 0.35a	58.80 ± 7.92a	54.41 ± 5.06a	1.29 ± 0.22a	21.49 ± 3.85a
**458**	C−	28.62 ± 2.75a	42.51 ± 2.38a	3.70 ± 0.36a	22.92 ± 3.55a	3.85 ± 0.88a	0.77 ± 0.13a	41.45 ± 5.24a	55.49 ± 7.09a	1.25 ± 0.21a	15.10 ± 5.99a
**458**	C+	26.86 ± 2.64a	42.74 ± 3.85a	3.52 ± 0.25a	21.85 ± 1.92b	4.22 ± 0.48a	0.65 ± 0.04a	40.94 ± 3.09a	53.83 ± 4.77a	1.22 ± 0.14a	14.87 ± 2.76a
**458**	CuNPs	28.46 ± 4.45a	45.57 ± 3.20a	3.69 ± 0.45a	29.02 ± 5.28a	4.39 ± 0.61a	0.67 ± 0.34a	38.46 ± 2.35a	52.34 ± 3.90a	1.13 ± 0.09a	17.08 ± 3.41a
**458**	CuONPs	28.94 ± 1.76a	43.05 ± 1.87a	3.60 ± 0.18a	21.49 ± 1.88b	4.43 ± 1.08a	0.44 ± 0.17a	36.29 ± 5.77a	47.41 ± 10.14a	1.02 ± 0.25a	15.13 ± 2.91a
**458**	CuSO4	25.97 ± 1.82a	40.41 ± 2.38a	3.30 ± 0.24a	26.72 ± 4.23a	4.73 ± 0.79a	0.44 ± 0.09a	36.00 ± 1.58a	45.36 ± 3.11a	1.03 ± 0.07a	17.64 ± 4.63a
**461**	C−	44.00 ± 5.00a	48.07 ± 6.12a	5.03 ± 0.95a	18.31 ± 2.17a	4.60 ± 0.27b	0.37 ± 0.07b	38.08 ± 7.39b	37.61 ± 10.01a	1.26 ± 0.28b	23.23 ± 4.91a
**461**	C+	42.89 ± 3.62a	48.92 ± 4.32a	5.14 ± 0.51a	17.55 ± 2.89a	4.64 ± 0.20a	0.62 ± 0.10a	48.34 ± 14.90a	47.35 ± 12.08a	2.39 ± 0.51a	18.48 ± 4.10b
**461**	CuNPs	49.37 ± 4.16a	57.09 ± 7.31a	5.67 ± 0.32a	18.10 ± 4.61a	5.02 ± 0.25a	0.96 ± 0.14a	60.90 ± 5.41a	58.05 ± 10.47a	3.17 ± 0.19a	15.91 ± 2.77b
**461**	CuONPs	50.54 ± 7.10a	53.83 ± 5.91a	5.79 ± 0.86a	19.57 ± 2.34a	5.32 ± 0.70a	0.84 ± 0.27a	60.00 ± 12.15a	60.73 ± 10.86a	3.06 ± 0.51a	26.31 ± 4.32a
**461**	CuSO4	44.57 ± 6.44a	48.39 ± 5.78a	4.96 ± 0.91a	20.97 ± 6.87a	3.98 ± 0.21b	0.94 ± 0.28a	39.48 ± 8.04b	35.60 ± 21.18a	2.85 ± 0.61a	24.31 ± 4.19a

*Data are presented as mean ± standard deviation (SD) of five independent biological replicates. Different lowercase letters within the same maize hybrid and biochemical parameter indicate significant differences among treatments according to Tukey's HSD test ($p < 0.05$). Priming agent: C−, deionized water; C+, culture liquid from untreated *Nostoc linckia* culture; CuNPs, culture liquid from Nostoc linckia cultivated with metallic copper nanoparticles; CuONPs, culture liquid from *Nostoc linckia* cultivated with copper oxide nanoparticles; $CuSO_4$, culture liquid from *Nostoc linckia* cultivated with $CuSO_4 \cdot 5H_2O$.
